# Kisspeptin and Neurokinin B: roles in reproductive health

**DOI:** 10.1152/physrev.00015.2024

**Published:** 2025-01-15

**Authors:** Kanyada Koysombat, Jovanna Tsoutsouki, Aaran H. Patel, Alexander N. Comninos, Waljit S. Dhillo, Ali Abbara

**Affiliations:** 1 Department of Investigative Medicine, Imperial College London, Hammersmith Hospital, London, United Kingdom; 2 Department of Endocrinology, Imperial College Healthcare NHS Trust, London, United Kingdom

**Keywords:** kisspeptin, neurokinin B (NKB), reproduction, puberty, KNDy

## Abstract

Kisspeptin and neurokinin B (NKB) play a key role in several physiological processes including in puberty, adult reproductive function including the menstrual cycle, as well as mediating the symptoms of menopause. Infundibular kisspeptin neurons, which co-express NKB, regulate the activity of gonadotropin releasing hormone (GnRH) neurons, and thus the physiological pulsatile secretion of GnRH from the hypothalamus. Outside of their hypothalamic reproductive roles, these peptides are implicated in several physiological functions including sexual behavior and attraction, placental function, and bone health.

Over the last two decades, research findings have considerably enhanced our understanding of the physiological regulation of the hypothalamic-pituitary-gonadal (HPG) axis and identified potential therapeutic applications. For example, recognition of the role of kisspeptin as the natural inductor of ovulation has led to research investigating its use as a safer, more physiological trigger of oocyte maturation in *in vitro* fertilization (IVF) treatment. Moreover, the key role of NKB in the pathophysiology of menopausal hot flashes has led to the development of pharmacological antagonism of this pathway. Indeed, Fezolinetant, a neurokinin 3 receptor antagonist, has recently received Food and Drug Administration (FDA) approval for clinical use to treat menopausal vasomotor symptoms.

Herein, we discuss the roles of kisspeptin and NKB in human physiology, including in the regulation of puberty, menstrual cyclicity, reproductive behavior, pregnancy, menopause, and bone homeostasis. We describe how perturbations of these key physiological processes can result in disease states and consider how kisspeptin and NKB could be exploited diagnostically, as well as therapeutically to treat reproductive disorders.

## Graphical abstract

**Graphical Abstract:**
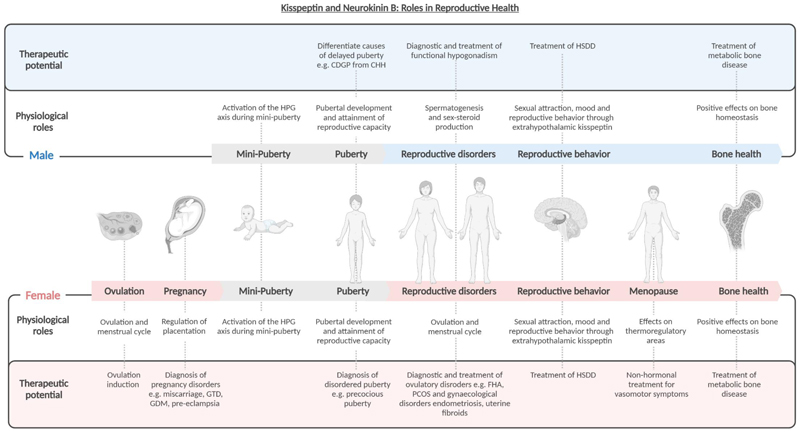

## Introduction

### The hypothalamic-pituitary-gonadal (HPG) axis

Successful reproduction requires sufficient energy and thus there is dynamic communication between homeostatic signals and hypothalamic GnRH function. The hypothalamic-pituitary-gonadal (HPG) axis is activated at puberty after a period of dormancy during childhood, marking the acquisition of reproductive capacity. At the apex of the HPG axis, kisspeptin neurons regulate pulsatile secretion of gonadotropin-releasing hormone (GnRH) from GnRH neurons into the hypophyseal portal circulation to stimulate pituitary gonadotrophs to release gonadotropins: i.e., luteinizing hormone (LH) and follicle stimulating hormone (FSH). In males, LH stimulates Leydig cells to produce testosterone, whereas FSH acts on Sertoli cells to support spermatogenesis. In females, the complex interplay between gonadotropins and sex-steroids are characteristic features of the ovarian cycle, encompassing folliculogenesis, ovulation, and luteal progesterone production.

Pioneering studies from Ernst Knobil were instrumental in establishing that pulsatile secretion of the decapeptide GnRH is requisite for the hypophysiotropic stimulation of pituitary gonadotrophs (1). In ovariectomized rhesus monkeys who were rendered GnRH deficient by lesioning the hypothalamic arcuate nucleus, chronic constant infusion of GnRH led to decline of serum gonadotropins, despite a brief initial flare of gonadotropins. By contrast, intermittent pulsatile administration of GnRH could stimulate gonadotropin secretion long-term. This concept was subsequently translated to women with GnRH deficient states such as functional hypothalamic amenorrhea (FHA) and hyperprolactinemia, whereby chronic intermittent administration of GnRH was able to normalize serum gonadotropin levels, restore menstrual cyclicity, and ovulation (2,3). The precise synchrony between GnRH and LH pulses, and the lack of LH pulses not preceded by GnRH pulses, confirmed the pulsatile nature of GnRH and in turn LH secretion (4,5). For many years, GnRH was believed to reside at the apex of the HPG axis, but GnRH neurons lack receptors for peripheral signals to modulate the HPG axis including estradiol (E2) or leptin receptors. Thus, the presence of the ‘GnRH pulse generator’, an upstream neuronal population that regulates GnRH neuronal function and could integrate peripheral metabolic and sex-steroid signals was posited.

In 2003, the seminal findings from two reports indicated that inactivating variants of the kisspeptin receptor gene (*KISS1R*) led to congenital hypogonadotropic hypogonadism (CHH) and a failure of the HPG axis (6,7). It was later discovered that these kisspeptin-expressing neurons in the infundibular nucleus (arcuate nucleus in animals), co-express neurokinin B (NKB), and regulate GnRH neuronal function, thus likely representing the ‘GnRH pulse generator’. Kisspeptin and NKB are now established to be hypothalamic neuropeptides that play a key role in regulating GnRH neuronal secretion.

In this review, we discuss the physiological roles of kisspeptin and NKB across the reproductive lifespan including during puberty, menstrual cyclicity, reproductive behavior, pregnancy, menopause, and bone homeostasis. We describe how perturbations of these key physiological processes can result in disease states, and we consider how kisspeptin and NKB could be exploited therapeutically, for example during *in vitro* fertilization (IVF), and the treatment for menopausal vasomotor symptoms.

## 0 Kisspeptin

### Discovery of kisspeptin and the kisspeptin receptor

The gene encoding kisspeptin, *KISS1*, and its transcript was first identified in 1996 through subtractive hybridization of micro-cell mediated transfer in melanoma and breast cancer cell lines as a metastasis-suppressor gene (8–10). The original complementary DNA (cDNA) designation, *KiSS1*, combined the nomenclature for putative Suppressor Sequences and paid homage to Hershey, Pennsylvania, where the gene was first discovered (10) and where the famous chocolate ‘*kisses*’ are produced. Using fluorescence in situ hybridization (FISH), the *KISS1* gene was mapped to the long arm of chromosome 1 (1q32-q41), consisting of four exons with two 5’ untranslated exons and two partially translated exons (11).

The cognate receptor for kisspeptin was discovered in an entirely unrelated context. Discovered in 1999, the cDNA clone encoding a G-protein coupled receptor, GPR54 in rats was noted to bear resemblance (40-50%) to the galanin receptor gene family (12). This gene mapped to chromosome 19q13.3 and had an open reading frame of 1191 base pair encoding a receptor consisting of 396 amino acids (12). In 2001, three separate groups working on orphan G-protein coupled receptors confirmed the human ortholog of the rat GPR54. Initially named hOT7T175 (13), AXOR12 (14) or GPR54 (15) the mRNA consisted of five coding exons, interrupted by 4 introns, and encodes a 398 amino acid protein in humans (cf. 396 amino acid in rats). In congruence with other G-protein coupled receptors, GPR54, now known as the kisspeptin receptor, has 7 hydrophobic putative transmembrane domains. Kisspeptin peptide was confirmed to be the cognate ligand of the GPR54 receptor (14) using peptides from human placental isolates (13,15).

**Figure 1:**
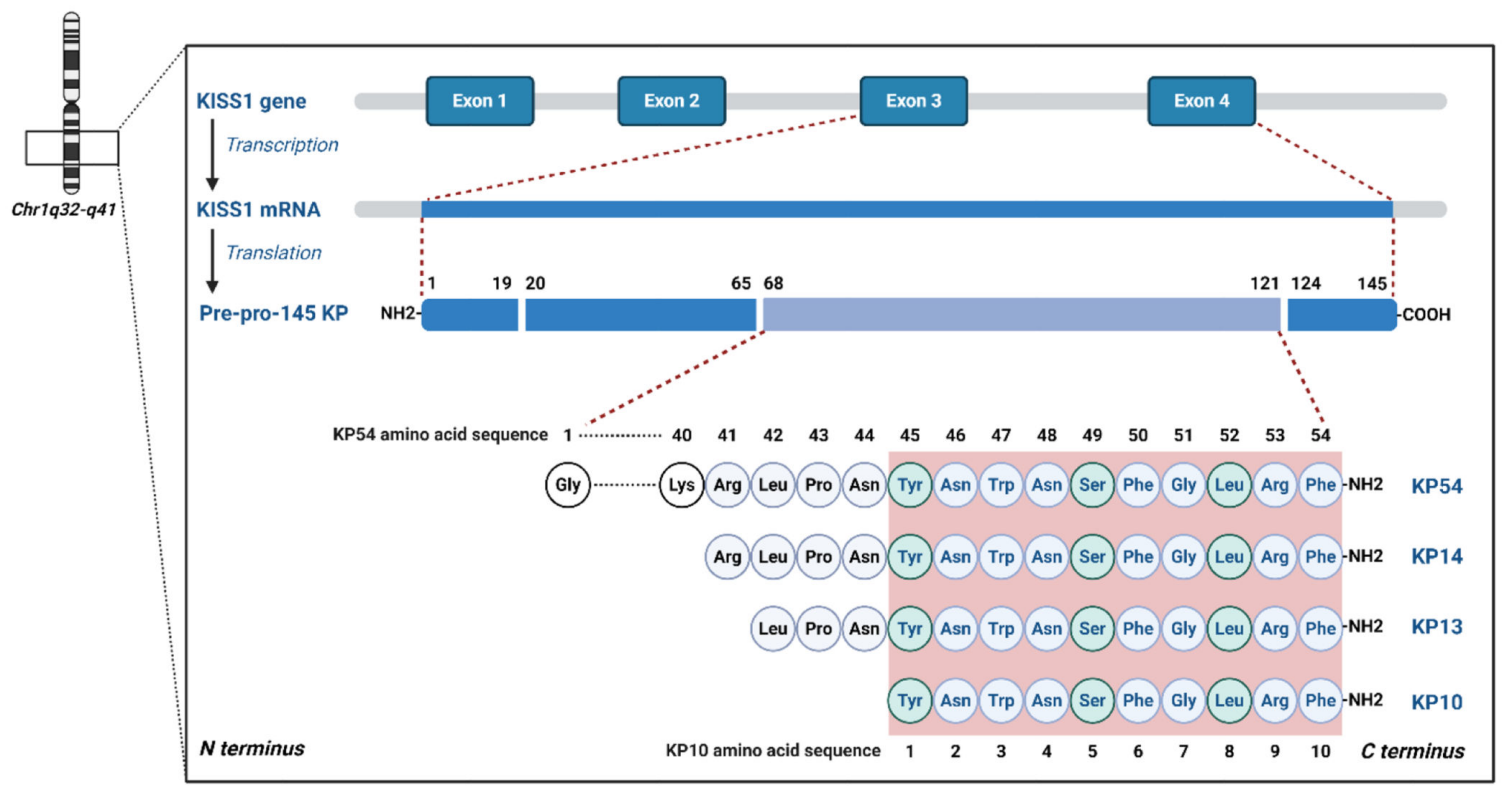
Kisspeptin gene and the major peptide forms of kisspeptin. *KISS1* gene mapped to the long arm of chromosome 1 (1q32-q41), consisting of four exons with two 5’ untranslated exons and two partially translated exons. *KISS1* encodes a 145 amino acid kisspeptin prepolypeptide which undergoes proteolytic cleavage into N-terminally truncated segments including kisspeptin-54 (KP54), kisspeptin-14 (KP14), kisspeptin-13 (KP13) and kisspeptin-10 (KP10); the suffix denoting their respective amino acid length. All native kisspeptin isoforms share a common C-terminal decapeptide Arg-Phe-NH_2_ motif characteristic of the RF-amide peptide family equivalent to kisspeptin-10. Figure created with BioRender.com.

*KISS1* encodes the 145 amino acid prepolypeptide now known as kisspeptin (previously termed metastin), which undergoes proteolytic cleavage into N-terminally truncated segments including kisspeptin-54, kisspeptin-14, kisspeptin-13 and kisspeptin-10; the suffix denoting their respective amino acid length (15,16). All native kisspeptin isoforms share a common C-terminal decapeptide equivalent to kisspeptin-10 including an Arg-Phe-NH_2_ motif characteristic of the RF-amide peptide family (13–15) (Figure 1). In binding- and functional-assays, kisspeptin-10 resulted in the highest potency in kisspeptin receptor activation, but the un-amidated C-terminal form had only very weak activity, indicating that the amidated RF-amide moiety of the C-terminal portion is required for binding and subsequent activation of the kisspeptin receptor (13–15). The kisspeptin receptor demonstrated selective activation by kisspeptin peptides, as other RF-amide peptides including neuropeptide FF and AF, RF-amide-like peptide-1 and 3 and prolactin-releasing peptide failed to activate the kisspeptin receptor (14,16).

### The physiological role of kisspeptin in reproductive health

The pivotal role of kisspeptin in reproductive physiology emerged in 2003 following two landmark papers reporting that inactivating variants in the GPR54 gene, now commonly known as the kisspeptin receptor (encoded by *KISS1R* gene in humans and *Kiss1r* in non-humans (17)), caused congenital hypogonadotropic hypogonadism (CHH) in human (6,7). Moreover, phenocopies were also achieved in mouse *Kiss1r* knockout models (18). In 2012, an inactivating variant in the gene encoding kisspeptin (*KISS1* in humans, *Kiss1* in non-humans (17)) was shown to also result in failure of pubertal initiation (19). Similarly, a *Kiss1* knockout mouse model also demonstrated lack of puberty, gonadal failure and infertility, albeit the phenotype was less severe than *Kiss1r* knockout models (20,21). By contrast, activating variants of both *KISS1* (22) and *KISS1R* (23) resulted in central precocious puberty (CPP). These rare cases and concordant animal models consolidated kisspeptin’s essential role as a regulator of the HPG axis.

As expected, kisspeptin-10 could not elicit an LH response in *Kiss1r* knockout models, however in these models, GnRH neuronal migration, GnRH synthesis and pituitary responsiveness to GnRH were preserved (7,21). Exogenous kisspeptin administration has since been demonstrated to stimulate gonadotropin secretion across multiple mammalian species through various administration modalities including both central (intracerebroventricular) and peripheral routes (intravenous, intraperitoneal and subcutaneous) (24–31). Moreover, kisspeptin had a direct effect on GnRH neurons in hypothalamic explants *ex vivo* (32). Mechanistically, kisspeptin’s ability to stimulate gonadotropin release is dependent on GnRH secretion, indeed administration of GnRH antagonist blocked its effect on gonadotropin secretion in animal models (24–26). Anatomically, kisspeptin immunoreactive fibers are in close apposition with GnRH neurons (30,33,34) and ~90% of GnRH neurons express *Kiss1r* mRNA in both juvenile and adult mice (24,35). Functionally, kisspeptin induced c-Fos expression in 86% of GnRH neurons (25), moreover electrophysiological studies demonstrated that kisspeptin directly depolarized GnRH neurons in murine brain slices (36). Altogether, these data suggest that kisspeptin acts upstream of GnRH and regulates GnRH neuronal function.

### Hypothalamic kisspeptin neuronal populations and the KNDy hypothesis

**Figure 2:**
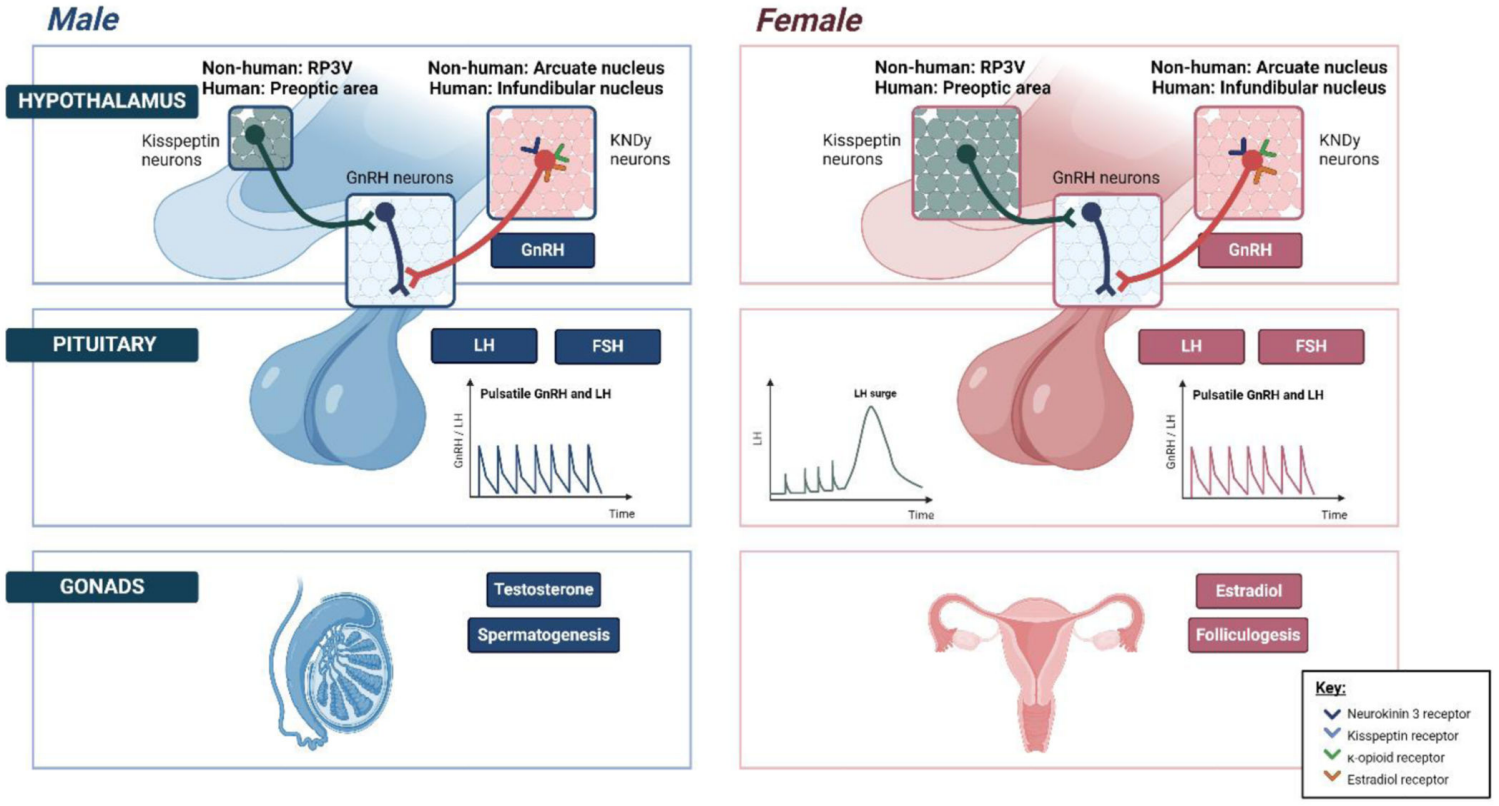
The hypothalamic-pituitary-gonadal axis in males and females. Distinct populations of kisspeptin neurons in the hypothalamus. Kisspeptin-expressing neurons are largely distributed in two discrete hypothalamic nuclei: the arcuate (analogous to the infundibular nuclei in humans) and the anteroventral periventricular nucleus (AVPV) in the rostral hypothalamus which extends into the preoptic periventricular nucleus (PeN) collectively termed the rostral periventricular area of the third ventricle (RP3V) (analogous to preoptic area in humans). RP3V kisspeptin neurons demonstrate female-dominant expression with 10-fold greater expression in females, whilst kisspeptin expression in the arcuate nucleus did not exhibit any discernible sexual dimorphism.

Kisspeptin-expressing neurons are largely distributed in two discrete hypothalamic nuclei: the arcuate (analogous to the infundibular nuclei in primates) and the anteroventral periventricular nucleus (AVPV) in the rostral hypothalamus, the latter extending into the preoptic periventricular nucleus (PeN) collectively termed the rostral periventricular area of the third ventricle (RP3V) (37). The RP3V neuronal population has female-dominant kisspeptin expression with a 10-fold sex difference, whilst the arcuate nucleus does not exhibit any discernible sexual dimorphism in rodents (37) (Figure 2).

Arcuate kisspeptin neurons co-express NKB and dynorphin collectively known as KNDy neurons; NKB stimulates whereas dynorphin inhibits kisspeptin release. These KNDy neurons (right panel) exhibit episodic activity that induce pulsatile GnRH, and in turn LH secretion, and are recognized to be the ‘GnRH pulse generator’ (at least in rodents). These arcuate kisspeptin expressing neurons are susceptible to E2 mediated negative feedback. By contrast, RP3V kisspeptin neurons (left panel) induce an LH surge through E2 mediated positive feedback to induce ovulation in females.

E2 receptors are associated with or embedded in the plasma membrane, E2 receptors are also located in the cytoplasm or the nucleus of target cells. However, within the limit of the schematic nature, this graphical depiction may not have fully captured this complex expression pattern.

AVPV, anteroventral periventricular nucleus; FSH, follicle stimulating hormone; GnRH, gonadotropin-releasing hormone; KNDy, kisspeptin, neurokinin B and dynorphin; LH, luteinizing hormone; PeN, periventricular nucleus; RP3V, rostral periventricular area of the third ventricle. Figure created with BioRender.com.

Arcuate kisspeptin neurons co-express neurotransmitters including NKB and dynorphin, and are thus termed ‘KNDy neurons’ (38). This expression pattern is highly conserved across various mammalian species including ewe (38), mouse (39), rat (40) and goat (41). In addition to appositions to GnRH neurons, interconnected networks of reciprocal KNDy-KNDy connections are capable of modulating and eliciting synchronized neuronal firing. KNDy neurons do not express *Kiss1r* but do express the NKB receptor (NK3R) and *κ*-opioid receptor. Whilst dynorphin/*κ*-opioid receptor agonist abrogated the slow excitatory postsynaptic potential, blockade of κ-opioid was able to revert this (42). By contrast, NKB and neurokinin 3 receptor agonist evoked stimulation of arcuate kisspeptin neurons (42). The regulation of KNDy neurons therefore occurs in an autocrine/paracrine manner with stimulatory NKB and inhibitory dynorphin signaling, whilst kisspeptin acts predominantly on downstream GnRH neurons (42). Indeed, administration of exogenous kisspeptin in patients with an inactivating variant of NKB signaling could restore LH pulse frequency, thus signifying the functional hierarchy in that NKB signaling functions upstream of kisspeptin (43). Interestingly, continuous infusions of kisspeptin can result in generation of pulsatile LH secretion in various species including in women with FHA (43–46). This could be due to kisspeptin amplifying and revealing previously undetectable low volume pulses that had still been generated by the GnRH pulse generator. Additionally, data suggests that GnRH neurons possess inherent pulsatility that could contribute to this curious clinical observation. Moreover, conflicting theories on the GnRH pulse generator are described further below.

The first systematic study in humans utilized hybridization histochemistry and computer-assisted microscopy to quantify and localize kisspeptin-expressing neurons in postmortem hypothalamic tissues (47). *KISS1* gene transcripts were identified predominantly within the hypothalamic infundibular nucleus (analogous to arcuate nucleus). KNDy neurons were demonstrated in the infundibular nucleus of post-mortem tissues from women (48) but the lack of colocalization in young men (49) suggests that age, sex and species differences may contribute to this variation. Moreover, the medial hypothalamic sections did not demonstrate a population of *KISS1* neurons in the RP3V as described in rodent models, although labeled neurons were noted to scatter sparsely within the medial preoptic area (47). Further immunohistochemical studies from postmortem human hypothalamic tissue corroborated these earlier findings, demonstrating that highest numbers of kisspeptin-54 immunoreactive cell bodies resided in the infundibular nucleus (50). Kisspeptin cell bodies were also observed in the rostral periventricular zone in female hypothalami and were hypothesized to anatomically represent the kisspeptin neurons of the RP3V observed in rodents, although the precise function of this neuronal population in human remains unclear (50). Recently, highly specific pre-prokisspeptin antibody-based immunohistochemical and immunofluorescent techniques enabled visualization of immunoreactive cell bodies in rostral hypothalamic sections in humans. These cell bodies were devoid of NKB, substance P, and cocaine and amphetamine-regulated transcript (CART) (51). Unlike kisspeptin neurons in the rodent RP3V, these kisspeptin neurons did not express neurotransmitters such as enkephalins, galanin and tyrosine hydroxylase (51). The identification of kisspeptin neurons in the human rostral hypothalamus and positive estrogenic regulation of this neuronal population challenge the paradigm that positive estrogen feedback is restricted to the mediobasal hypothalamus in primates (51).

### GnRH pulse generator

The concept of the ‘GnRH pulse generator’ was coined in the 1980s, positing that pulsatile LH secretion is controlled by a hypothalamic pulse generator that regulates GnRH neurons. Selective lesioning of the arcuate nucleus resulted in cessation of gonadotropin secretion whilst function of the basal thyroid, adrenocortical, and growth hormone axes were preserved (52). By comparison, complete deafferentation of the mediobasal hypothalamus (MBH) with sparing of the arcuate nucleus did not impact the pulsatile rhythm of gonadotropins in rats (53) or monkeys (54), and the positive feedback action of E2 on gonadotropin release was also preserved. Early electrophysiological studies recorded from the vicinity of the arcuate nucleus provided evidence that multiunit electrical activity (MUA) volleys were invariably associated with initiation of LH secretion (55). These anatomical and electrophysiological studies therefore suggested that the arcuate region was the primary structure mediating the hypothalamic control of gonadotropin secretion in the rhesus monkey and suggested that the pulse generator exists in this area, thus initially named the ‘arcuate oscillator’.

Whether the pulse generator was inherent within GnRH neurons or whether this was mediated extrinsically via afferent neurons located in the arcuate nucleus was debated at the time. Evidence for the concept that the pulsatility was intrinsic to GnRH neurons stem largely from *in vitro* immortalized (56) and subsequently embryonic GnRH cell lines (rhesus monkeys (57); rats (58); sheep (59); mouse (60)). These cell lines demonstrated GnRH pulsatility profile and interpulse frequency reminiscent of those observed in castrated rodents suggesting that synchronization could be mediated cell-to-cell or through a diffusible mediator in a paracrine manner. However, the diffuse localization of GnRH perikarya *in vivo* questions the applicability and translatability of these findings. Furthermore, the ability of isolated median eminence explants devoid of GnRH cell bodies to also elicit GnRH pulsatility, suggests that synchronization between GnRH cell bodies may not be necessary to synchronize pulsatile GnRH secretion (61).

Variation in the frequency of pulsatile GnRH secretion leads to differential LH and FSH secretion (62,63). In the early follicular phase, low amplitude pulses occur approximately every 1-1.5hrs, with transitions to high amplitude pulses every 3-4hrs during most of the luteal phase (61). This variation is purported to be secondary to homeostatic cues including E2. E2 predominantly exerts its action via estrogen receptor-alpha (ERα), however, as GnRH neurons lack ERα as well as androgen and progesterone receptors, sex-steroid feedback is therefore likely mediated indirectly via an intermediary neuronal population (64). Together this evidence suggests that the GnRH pulse generator is likely to be extrinsic to the GnRH neurons.

In 2017, optogenetic approaches captured near-perfect correlation between pulsatile LH secretion (proxy of GnRH secretion) and brief repetitive episodes of elevated calcium within the arcuate neuronal population (65). Selective activation and inhibition of these arcuate KNDy neurons could stimulate and suppress pulsatile LH secretion respectively, thus providing functional evidence that arcuate KNDy neurons are indeed the GnRH pulse generator, at least in rodents (65). Recently, fiber photometry and *in vivo* calcium recordings of KNDy cells demonstrated that synchronized activity of KNDy cells preceded LH pulses (66). Studies of KNDy cellular activity at a single-cell level provided the required granularity to reveal that synchronized episodes in KNDy cells occur in a predictable temporal order with ‘leader cells’ capable of initiating episodic LH pulses (67). The tight temporal relationship between LH and corresponding synchronized KNDy cell activity in rodent studies supports the hypothesis that KNDy neurons are important components of the GnRH pulse generator.

GnRH neurons have a bipolar morphology consisting of the soma and proximal dendrites with few dendritic processes possessing blended dendritic- and axonal-like properties termed ‘dendrons’ (68). Morphologically, these dendrons are interconnected, receive shared inputs from afferent neurons, with KNDy neurons forming appositions at the distal dendrons to enable synchronized GnRH secretion into the portal vasculature (69). Surprisingly, using expansion microscopy it was shown that unlike the classical synapses observed between KNDy neurons, GnRH somata and proximal dendrites; KNDy neurons make non-synaptic appositions with GnRH neuronal dendrons and activate them via short-distance volume transmission (69). Through selective inhibition of proximal and distal GnRH neuronal dendritic compartments, recent chemogenetic studies demonstrated that the distal dendritic zones are important during both pulsatile secretion and generation of GnRH/LH surge, whilst the soma-proximal dendritic compartment appears to be critical for the generation of the GnRH/LH surge (70).

### Extrahypothalamic kisspeptin neuronal populations

In addition to the hypothalamic kisspeptin neuronal populations, *Kiss1/KISS1* mRNA was also detected in several areas of the central nervous system (CNS) (including the pituitary gland, basal ganglia, amygdala, substantia nigra and hippocampus), the placenta, pancreas, and bone. *KISS1R* mRNA is expressed in similar regions as *KISS1* mRNA, abundantly expressed in the placenta, pituitary gland, spinal cord, and pancreas with lower expression in extrahypothalamic brain regions and various tissues such as the stomach and small intestine (14,15).

Whilst the hypothalamic action of kisspeptin is well recognized, the expression of kisspeptin receptor and its cognate ligand in the pituitary suggests a potential direct action of kisspeptin at the level of the pituitary gland. Specifically, the expression of *Kiss1* and *Kiss1r* in the pituitary is differentially regulated by the sex-steroid milieu; *Kiss1* expression decreased following ovariectomy but pre-treatment with E2 was able to prevent this, whilst *Kiss1r* expression increased following ovariectomy, but this effect was also negated with E2 treatment (71). In rhesus monkeys, kisspeptin-positive cells were observed in the intermediate and anterior lobe of the pituitary gland (72) and push-pull perfusate samples from the median eminence of pubertal monkeys indicate significant kisspeptin increments in association with GnRH (73). Similarly, the presence of kisspeptin in the ovine hypophysial portal blood also supported a potential role of kisspeptin on the anterior pituitary gland (74).

Despite the anatomical and functional evidence for possible direct action of kisspeptin at the level of the pituitary, the importance of the anterior pituitary in mediating kisspeptin action remains contestable. For example, even though low levels of kisspeptin are detected in the hypophysial portal circulation, no temporal rise in kisspeptin levels were seen with the E2-induced GnRH/LH surge in ewes (74). Surgical disconnection of the hypothalamic-pituitary unit abolished the LH rise following kisspeptin stimulation (74) similar to that observed following pre-treatment with GnRH antagonists (24,35). Most recently selective *Kiss1r* knockout in the pituitary gonadotrophs using a PKiRKO mouse model which achieved an 88% and 64% reduction in *Kiss1r* mRNA in the pituitary of male and female mice, respectively, have been used to delineate the contribution of the pituitary kisspeptin signaling (75). Phenotypically there were no differences in pubertal timing, gonadal weight and basal gonadotropin levels observed in the PKiRKO models, which although do not negate the potential direct pituitary actions of kisspeptin, suggests that kisspeptin signaling at the level of the pituitary does not play a major role in the control of the HPG axis(75).

Finally, reproductive tissues including the ovaries express kisspeptin/KISS1R and NKB/NK3R locally, and their putative roles will be further described in the subsequent section. Altogether, these findings strongly suggested the potential involvement of the kisspeptin system in the control of diverse physiological systems (76), which are explored further in the relevant subsequent sections below.

## 1 Puberty

Puberty is characterized by sexual maturation and acquisition of reproductive capacity following activation of the HPG axis from its quiescent prepubertal state (77). In most mammals, during late prenatal or early postnatal life, a period of transient activation of the HPG axis known as ‘*mini puberty*’ believed to be important for priming reproductive organs is followed by period of relative quiescence until the onset of puberty (77,78). The resurgence of pulsatile GnRH release is recognized as the key neuroendocrine initiator central to the onset of puberty (79).

**Figure 3:**
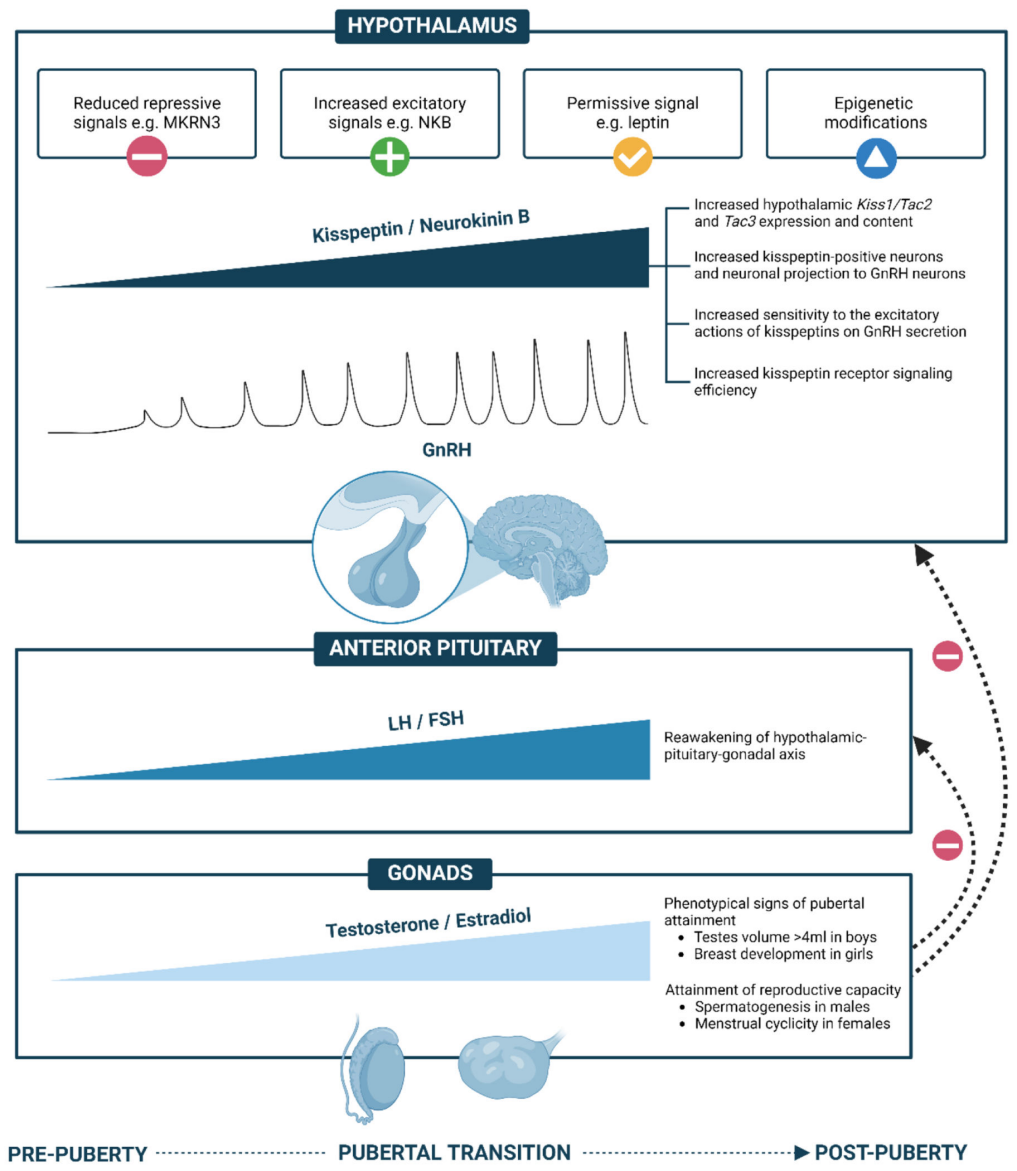
The physiological role of kisspeptin and neurokinin B in puberty. Puberty marks the reactivation of the hypothalamic-pituitary-gonadal axis from its quiescent pre-pubertal state. Neuroendocrine changes governing the timing of this process include increased excitatory, lowered inhibitory signals and permissive signals that integrate genetic, environmental, and metabolic factors on GnRH neurons. Increased kisspeptin expression, increased number of *Kiss1* neurons and their projections to GnRH neurons, enhanced sensitivity to the excitatory action of kisspeptin and increased kisspeptin receptor signaling efficiency across the pubertal transition provide evidence for kisspeptin’s role in the physiology of puberty.

The re-awakening of the gonadotropic axis and attainment of reproductive capacity during puberty involves the complex interplay of enhanced excitatory, lowered inhibitory signals and permissive signals that integrate genetic, environmental, and metabolic factors on GnRH neurons (Figure 3). In recent years, through studies in patients with disordered puberty i.e., precocious or delayed/absent puberty, key neuroendocrine players that contribute to regulating hypothalamic function have enhanced our understanding of the physiological regulation of puberty and the HPG axis.

FSH, follicle stimulating hormone; GnRH, gonadotropin-releasing hormone; LH, luteinizing hormone; MKRN3, makorin RING finger protein 3; NKB, neurokinin B. Figure created with BioRender.com.

### The physiological roles of kisspeptin in puberty

Data from electrophysiological studies mark kisspeptin as one of the most potent excitatory stimuli to GnRH neurons yet discovered (80). The proposed involvement of kisspeptin in the timing of puberty onset was based on loss-of-function variants in kisspeptin signaling leading to delayed or absent puberty, whilst gain-of-function variants led to precocious puberty (6,7,19,22,23).

Expression of hypothalamic *Kiss1* mRNA/kisspeptin content in rodents and rhesus monkeys increases throughout the pubertal transition (28,81). Intriguingly, whilst the number of *Kiss1* expressing cells increases by 7-fold in the RP3V, *Kiss1r* expression on GnRH neurons appeared unaltered across puberty (35) suggesting that increased RP3V kisspeptin neuronal projection to GnRH neurons may contribute to reactivation of GnRH neurons peripubertally. Indeed, appositions between kisspeptin fibers and GnRH neuron somata was first apparent at postnatal day 25 and increased throughout pubertal development in rodents (37). Thus, kisspeptin appears to be an important instigator of pubertal onset and reactivation of the HPG axis.

Central kisspeptin administration to prepubertal male and female rats could induce GnRH release, increasing low prepubertal LH levels to adult levels (81). However, when compared to adult mice with robust LH responses to kisspeptin, juvenile mice required higher doses of kisspeptin stimulation to elicit an LH response (35). Likewise, electrophysiological studies have demonstrated that GnRH neurons acquire sensitivity to the excitatory actions of kisspeptin during the pubertal transition, with least GnRH neuron depolarization seen in juvenile mice and maximum depolarization seen in adult mice (35). Functionally, chronic central kisspeptin administration to prepubertal female rats resulted in a precocious pubertal phenotype, evidenced by premature vaginal opening, increased uterine weight, LH and E2 levels (82). Conversely, central infusion of the kisspeptin antagonist, p234, to peripubertal female rats led to a marked delay in pubertal timing with significantly delayed vaginal opening, reduced ovarian and uterine weight (83). Similarly, kisspeptin-10 administration resulted in significant LH and testosterone increase in boys at Tanner stage V and adult men but not in boys at Tanner stages I-IV (84). Female adult rats demonstrated failure to sustain LH secretion after 48hrs of chronic kisspeptin-10 infusion in keeping with desensitization (85). In contrast, at the time of puberty, LH concentrations were persistently elevated even at 7 days after constant kisspeptin-10 administration, suggesting persistent stimulation (85). All in all, increased numbers of *Kiss1* neurons and their projections to GnRH neurons, enhanced excitatory action of kisspeptin and improved kisspeptin receptor signaling efficiency across the pubertal transition provide further evidence for kisspeptin’s role in the instigation and maintenance of puberty (Figure 3).

In 2011, a series of toxin-based genetic studies achieved over 90% targeted ablation of neurons expressing kisspeptin and kisspeptin receptors (86). In the absence of kisspeptin or kisspeptin receptor expressing neurons, mice exhibited reduced ovarian size, however these mice did not exhibit impaired pubertal maturation or fertility suggesting that kisspeptin neurons are needed for full gonadal maturation but may be dispensable with regards to the timing of puberty onset (86). By contrast, ablation of kisspeptin expressing neurons in adult female mice led to acyclicity and infertility, however this phenotype was not replicated by conditional ablation of kisspeptin receptors (86). The authors concluded that the phenotypic discrepancies between congenital and conditional ablation of kisspeptin-expressing neurons may be due to developmental compensation; thus, ablation of kisspeptin-expressing cell in adulthood appears to preclude activation or formation of these alternative reproductive circuits (86). Furthermore, following ablation, the remaining 7% of the GnRH neuronal population (i.e. the non-kisspeptin receptor expressing GnRH population) was sufficient to mediate reproductive function suggesting considerable redundancies in the HPG axis (86) consistent with prior data (87). Further exploration of the plasticity and redundancy of the kisspeptin system in the reproductive axis demonstrated that a 95% reduction in *Kiss1* transcript levels in mice still allowed a normal reproductive phenotype in male mice, whereas females are subfertile, suggesting that females require higher levels of kisspeptin expression for reproductive capacity (88). Recently, using female global *Kiss1* knockout rats with no gonadotropin activity, reintroduction of kisspeptin to only 20% of arcuate KNDy neurons was able to rescue folliculogenesis and pulsatile LH release (66). Therefore, akin to GnRH, the kisspeptin system displays considerable redundancy with only a limited number of functional KNDy cells required to generate sufficient LH pulses to maintain downstream activation of the HPG axis, with this being greater in females than males.

### The physiological roles of NKB in puberty

NKB and dynorphin are neuropeptides co-expressed in arcuate KNDy neurons. These neuropeptides act in a concerted auto/paracrine manner to regulate the pulsatile GnRH release, and represent the ‘GnRH pulse generator’. NKB, encoded by *Tac2* in rodents and *TAC3* in humans, binds preferentially to the neurokinin 3 receptor (NK3R) encoded by *Tac3r* in rodents and *TACR3* in humans. In 2009, seminal papers reported that inactivating variants of *TAC3* and *TACR3* led to hypogonadotropic hypogonadism in humans (89,90) and in female mice (91), identifying the critical role of NKB for normal pubertal timing.

Hypothalamic expression of *Tac2* and *Tac3r* mRNA increases progressively through postnatal ages with maximal expression seen at time of puberty onset for *Tac2*. In contrast to *Kiss1*, which demonstrates increased expression within the RP3V postnatally until puberty with no discernible change within the arcuate nucleus, *Tac3r* expression was significantly increased within the arcuate (92). Central and peripheral administration of NKB, and the NK3R agonist, senktide, have been shown to stimulate GnRH neurons and downstream LH release in several mammalian species, likely through inducing kisspeptin release (93–95). Senktide could also stimulate LH release in pre-pubertal rodents and the amplitude of LH response increased with advancing post-pubertal age (92). Conversely, chronic infusion of an NK3R antagonist during puberty led to mildly decreased LH levels and delayed vaginal opening in female mice (92).

However, unlike disruption in kisspeptin signaling, the reproductive phenotype of deficient NKB signaling is notably milder. For instance, humans harboring inactivating variants in *TAC3/TACR3*, could subsequently proceed to achieve HPG axis activation in adulthood, a phenomenon termed ‘reversal’ (96). In rodent models, *Tac2*^-/-^ knockout male mice exhibit no delay in sexual maturation or fertility however, in contrast, *Tac2*^-/-^ females had profound delays in sexual maturation and initial abnormalities in estrous cycles which then recovered during adulthood and these females were ultimately fertile (91). *Tac3r* null mice demonstrated normal markers of sexual maturation, however males had lower testicular weight and females had lower uterine and abnormal estrous cycles with prolonged time spent in diestrus, nonetheless, these mice were fertile (97). Overall, this suggests that NKB signaling is important in early sexual development and timing of puberty.

### The physiological roles of kisspeptin and NKB in integrating metabolic signals in puberty

The tempo of puberty is orchestrated by the dynamic interactions between genetic and environmental factors. As both puberty and reproduction are energy demanding physiological processes, endogenous signals reflective of the body’s energy availability are essential for successful reproduction and subsequent lactation. Metabolic perturbations, both under- and over-nutrition, can affect pubertal onset. Leptin, an anorexigenic adipokine secreted by white adipose tissue reflective of total body energy stores (98,99), is a permissive signal requisite for healthy GnRH neuronal function. Leptin has an unequivocal role in puberty wherein leptin deficient mice and humans have absent puberty and are infertile (100).

Changes in leptin levels reflective of energy availability are postulated to impact on kisspeptin and NKB expression/function. Through manipulating postnatal feeds, pubertal timing has been shown to be sensitive to early nutritional availability. Overfeeding resulted in higher levels of leptin, *Kiss1* mRNA expression and earlier vaginal opening whilst subnutrition led to lower levels of leptin and *Kiss1* mRNA paralleled by delayed vaginal opening (101). In prepubertal rats, chronic administration of kisspeptin was sufficient to stimulate gonadotropin and estrogen secretion and restore vaginal opening despite reductions in *Kiss1* mRNA expression induced by caloric restriction (102). Continuous kisspeptin-10 infusion to underfed pubertal female rodents with marked hypo-leptinemia significantly increased serum LH, uterine weight and restored vaginal opening in >62% of the animals over a 7-day treatment period despite no change in body weight (85). In contrast, chronic leptin infusion, which led to further body weight reduction, failed to persistently elevate gonadotropins and only rescued vaginal opening in 25% of animals (85) consolidating kisspeptin’s key role in maturation of the HPG axis.

Likewise, rodent models subjected to caloric restriction, leading to >25% reduction in body weight demonstrated decreased arcuate *Tac3r* mRNA expression. LH response to senktide in caloric restriction model was enhanced in comparison to age-matched controls fed *ad libitum*, with some caloric restricted animals demonstrating complete vaginal opening (91). These findings reaffirm the intricate links between metabolic status and reproductive health mediated through kisspeptin and NKB expression/function, culminating in the downstream effects on GnRH output and the resultant reproductive phenotypes.

The effect of leptin on kisspeptin neurons is likely to be mediated indirectly through an intermediary neuronal population as selective deletion of leptin receptor from kisspeptin neurons (103) in mice had no effect on fertility. Arcuate agouti-related peptide (AgRP) neurons, which co-express neuropeptide Y (NPY) and GABA; and pro-opiomelanocortin (POMC) neurons which co-express cocaine- and amphetamine-regulated transcript (CART), are likely candidates given their abundant expression of leptin receptors as well as their well-recognized role in energy homeostasis. In the hypoleptinemic state, orexigenic AgRP/NPY neurons are hyperactivated (104), whilst anorexigenic POMC neurons are suppressed (105).

The neuropeptides AgRP secreted by AgRP neurons, and alpha-melanocyte stimulating hormone (α-MSH), the main secretory product of POMC neurons, are respective antagonist and agonist of the melanocortin 3 receptor (MC3R) and melanocortin 4 receptor (MC4R) (106). These neuropeptides have been shown *in vitro* to act directly on GnRH and kisspeptin neurons (106).

AgRP neurons form physical connections with arcuate and RP3V kisspeptin neurons (107). In female mice lacking leptin receptors, AgRP neuronal ablation was able to restore reproductive function (108). Furthermore, selective AgRP neuron-specific rescue of leptin receptors in leptin receptor null mice could partially or fully restore reproductive function despite persisting metabolic effects (109) thus demonstrating AgRP neuron’s role in integrating the metabolic effects of leptin and reproductive phenotype.

Neuroanatomically, POMC neurons send projections to GnRH cell bodies and terminals (106) and immunoreactive α-MSH fibers were identified in close apposition to *Kiss1* cell bodies of pubertal rats (110). Chronic MC3R and MC4R blockade in peripubertal female rat induced significant suppression of arcuate *Kiss1* resulting in delayed puberty. Moreover, in *Kiss1r* knockout rodent model, activation of MC3R and MC4R failed to stimulate an LH response (111). These preclinical studies suggest that the α-MSH/melanocortin system acts upstream and is dependent of kisspeptin to exert its effect on the reproductive phenotype. In corroboration with preclinical data, a homozygous loss of function variant in MC3R in humans was associated with reduced linear growth, reduced lean mass, raised body mass index (BMI), and delayed puberty (112,113).

### Epigenetic modulation of kisspeptin and NKB in the regulation of puberty

Epigenetic regulation governs gene expression through DNA methylation and hydroxymethylation, post-translational modifications of histones, and non-coding RNAs such as microRNAs (miRNA). DNA methyltransferases (DNMTs) and ten-eleven translocation (TET) enzymes mediate DNA methylation and demethylation, respectively (114). The balance between methylated and demethylated DNA influences chromatin structure, which in turn determines transcriptional activity through conformational changes of chromatin (115). Histones are key structures in nucleosomes, post-translational modifications of histones through processes including acetylation, methylation, phosphorylation, ubiquitination and sumoylation, can thereby influence transcriptional activity (114). For example, the role of epigenetic silencers and activators in fine-tuning pubertal timing has been noted through activation or repression of *Kiss1* expression. Prepubertally *Kiss1* expression is low, in part due to expression of Polycomb Group (PcG) epigenetic silencers, whereas during pubertal progression, *Kiss1* expression is enhanced following recruitment of epigenetic activators, such as the Trithorax group to the promoter region of *Kiss1* (116). This subsequently affects chromatin structure (i.e., epigenetic silencer induces compaction whereas activator induces open conformation), which affects access of the transcriptional machinery and thereby final *Kiss1* gene expression (116).

Specifically, within the PcG system, hypothalamic mRNA expressions of *Cbx7* and *Eed*, two PcG genes required for PcG action, decrease at the initiation of puberty. Arcuate Kiss1 neurons co-express both *Cbx7* and *Eed7* gene; indeed, the increase in *Kiss1* expression observed during the pubertal transition was accompanied by eviction of EED from *Kiss1* promoter, highlighting a putative mechanism of PcG mediated repression in the regulation of pubertal timing (117). Conversely, mixed-lineage leukemia 1 (MLL1) and 3 (MLL3), two members of the Trithorax group, have been shown to counteract the repressive actions of PcG by facilitating the configuration chromatin changes from repressive to permissive by acting at the *Kiss1* and *Tac3* promoter regions (118).

Epigenetic modifications also provide a potential link between nutritional status and pubertal development. Sirtuin 1 (SIRT1), an energy-sensing deacetylase, is abundantly expressed in arcuate/MBH Kiss1 neurons (119). Hypothalamic SIRT1 content decreased during the pubertal transition, which coincided with increased *Kiss1* and *Tac3* expression. Overnutrition leads to earlier pubertal development in rodent models and is associated with a reduction in *SIRT1* and elevation of both *Kiss1* and *Tac3* expression; whilst the converse phenotypic and expression profiles are observed in undernutrition; moreover, transgenic overexpression of *SIRT1* led to delayed pubertal maturation (119). Mechanistically SIRT1 have been shown to repress Kiss1 expression through interaction with the PcG complex at the *Kiss1* promoter region and acts synergistically with EED to induce a repressive chromatin configuration and thereby reduces *Kiss1* transcription (119).

Several hypothalamic zinc finger genes (ZNF) are downregulated in the MBH during the juvenile-pubertal transition in monkeys. Notably *GATAD1* and *ZNF573* overexpression delayed pubertal onset and GATAD1 have been shown to repress *KISS1* and *TAC3* promoter activity (120). Another zinc finger protein, initially termed zinc finger protein 127 (*ZNF127*), now renamed makorin RING finger protein 3 (MKRN3) is recognized as a puberty-suppressing factor acting upstream of GnRH secretion encoded by the gene located in the Prader-Willi syndrome critical region (121). MKRN3 belongs to a family of E3 ubiquitin ligases involved in the ubiquitination process important in regulation of protein degradation (121). Whole-exome sequencing in patients from 15 families with central precocious puberty demonstrated loss-of-function variants in *MKRN3* (121). Moreover, in rodent models, *Mkrn3* mRNA was expressed in the arcuate nucleus with heightened juvenile expression and with striking reduction immediately prior to puberty (121). This pubertally regulated transition was also replicated in female rats and rhesus monkeys irrespective of the sex-steroid milieu (122). *MKRN3* is most strongly expressed in the ventromedial and arcuate nuclei and colocalizes with arcuate kisspeptin-expressing neurons (122). Mechanistically, MKRN3 selectively inhibits *KISS1* and *TAC3* promoter activity, thereby inhibiting kisspeptin and NKB expression without affecting the promoter region of *PDYN* (which encodes prodynorphin) (122). Lastly, MKRN3 represses *KISS1* and *TAC3* gene promoter activities through its action as an E3 ubiquitin ligase with reduced activity in pathogenic variants affecting the RING finger domain of the protein (122) and may also target neuropeptides of Kiss1 neurons to ubiquitination and degradation pathways (123). Kisspeptin therefore mediates the final common pathway downstream of MKRN3 to determine pubertal timing.

Finally, non-coding mRNAs such as miRNAs, largely through translation suppression or RNA degradation, have been shown to be important in the regulation of puberty. The RNAase III enzyme, Dicer, is important in the final step of mature miRNA biosynthesis. Selective inactivation of Dicer in GnRH neurons led to central hypogonadism and failure of pubertal completion in mice through involvement of miR-200/429 family and miR-155 (124). Likewise female mice engineered to lack miRNA synthesis in *Kiss1* neurons failed to complete puberty and attain fertility (125).

### Disorders of puberty

Delayed puberty is defined as the absence of testicular enlargement (testicular volume < 4ml, Tanner stage 2) in boys and breast development in girls (Tanner stage 2) at an age that is 2 standard deviations later than the population mean, traditionally defined as the age of 14 years in boys and 13 years in girls (126). Biochemically, most boys (95%) and girls (75-85%) with delayed puberty will have low levels of sex-steroids and inappropriately normal or low levels of gonadotropins consistent with hypogonadotropic hypogonadism (127). Constitutional delay of growth and puberty (CDGP) is the most common cause of delayed puberty, which affects 60-80% of boys and 30-55% of girls with biochemical hypogonadotropic hypogonadism (127). CDGP represents a variant of the normal spectrum of pubertal timing, and affected adolescents will proceed through puberty spontaneously without treatment albeit delayed to their peers (126–129). An important but less common cause of delayed puberty with similar presentation and biochemical profile to CDGP is CHH. CHH is caused by genetic variants causing impaired hypothalamic GnRH neuronal migration or function, affecting 10% of younger adolescent boys and 10-20% of girls (127). Whilst CDGP can usually be managed conservatively, patients with CHH benefit from treatment with pubertal induction to safeguard future reproductive, sexual, bone, metabolic and psychological health (130). Timely and accurate distinction between CDGP and CHH is challenging; due to the overlapping clinical features, biochemical profiles and the absence of a ‘gold standard’ diagnostic test (131).

Precocious puberty is defined as sex-hormone production or exposure occurring earlier than that which is expected for gender, ethnicity and race; typically with female preponderance (132). In girls, this is defined as the onset of breast development before the age of 8 years and in boys as increased testicular volume (>4ml) before the age of 9 years, accompanied by acceleration of linear growth and bone age. Precocious puberty can be classified as GnRH-dependent or GnRH-independent processes. GnRH-dependent or central precocious puberty (CPP) results from the premature activation of the HPG axis by CNS abnormalities, whilst GnRH-independent or peripheral precocious puberty results from the unregulated gonadal production of sex-steroids (132).

### Kisspeptin as a diagnostic test in disorders of puberty

#### Delayed puberty

The ability of kisspeptin to directly stimulate hypothalamic GnRH release offers an opportunity to use it as a test to evaluate hypothalamic function (133–136). Kisspeptin administration has been utilized as an *in vivo* interrogator of the GnRH neuronal function in CHH (133–136). As hypothalamic GnRH neuronal migration/secretion/function is impaired in CHH, patients with CHH have minimal gonadotropin response to kisspeptin (133–136). Intravenous boluses of kisspeptin-10 or kisspeptin-54 result in lower LH levels than in healthy controls in both adult and pediatric cohorts (133–136).

In adults, kisspeptin-54 led to maximal LH rise of 12.5 IU/L in eugonadal men compared to 0.4 IU/L in men with CHH (135). When compared to a GnRH test, kisspeptin-54 more accurately differentiated men with CHH from eugonadal men, with no overlap between the two cohorts (area under receiver operating characteristic curve (AUROC) kisspeptin-54: 1.0, 95% confidence interval (CI) 1.0–1.0; GnRH: 0.88, 95% CI 0.76–0.99) (135). Within the CHH cohort, LH rises after kisspeptin-54 were also significantly lower in those with anosmia as compared to normosmic patients. Likewise, patients with CHH who had identified pathogenic/likely pathogenic variants in CHH genes had even lower LH rises after kisspeptin compared to other men with CHH (135). Furthermore, in a small cohort of patients with sustained CHH reversal, response to kisspeptin-10 was regained suggesting that a kisspeptin test could serve as a useful test for current hypothalamic function (133).

**Figure 4:**
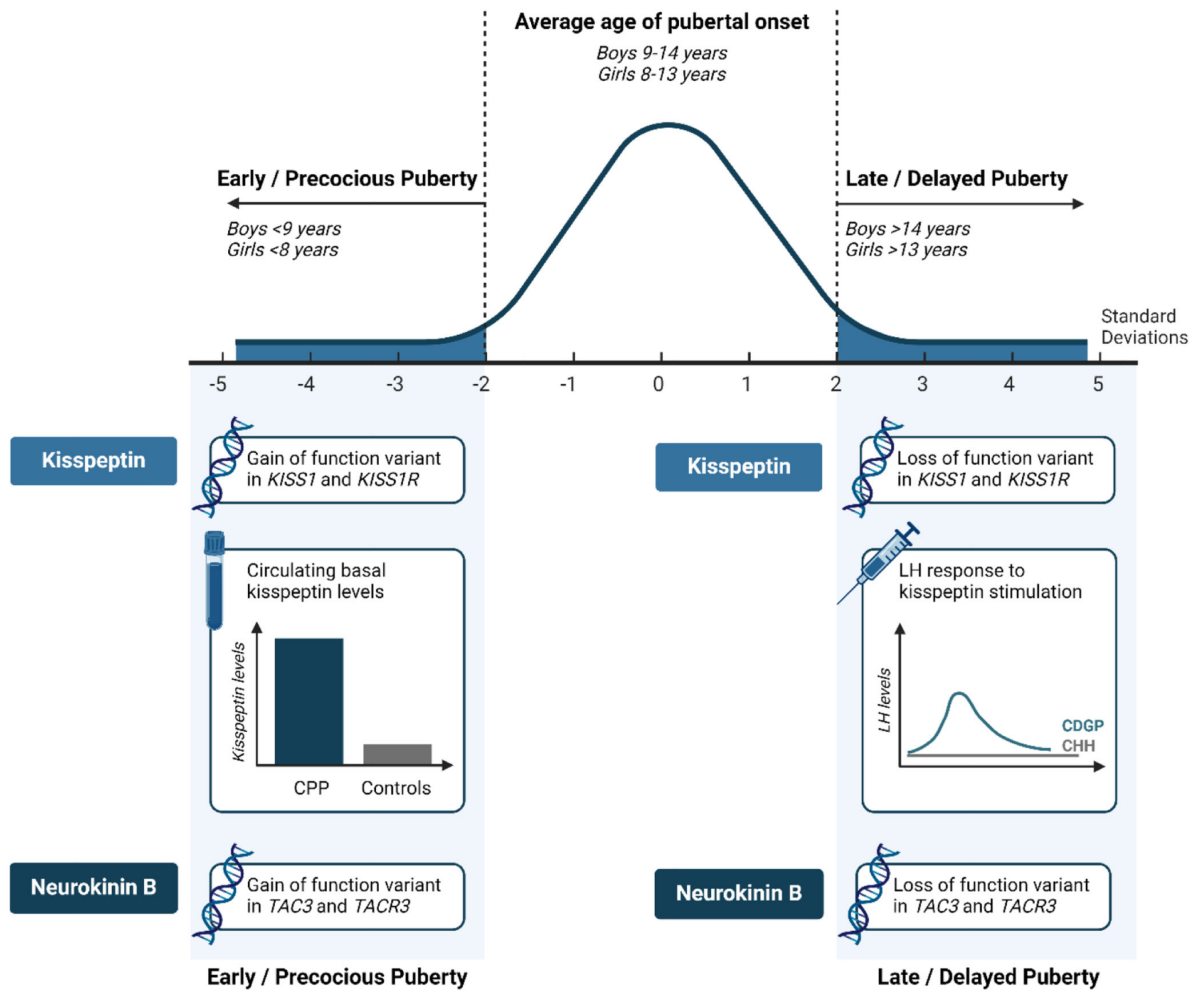
Kisspeptin and neurokinin B in disordered puberty. Delayed puberty is the absence of testicular enlargement (testicular volume < 4ml, Tanner stage 2) in boys and breast development in girls (Tanner stage 2) at an age that is 2 standard deviations later than the population mean (traditionally defined as the age of 14 years in boys and 13 years in girls). Precocious puberty is defined as sex hormone production or exposure occurring earlier than that which is expected for gender, ethnicity, and race; traditionally defined as the onset of breast development before the age of 8 years and in boys as increased testicular volume before the age of 9 years, accompanied by acceleration of linear growth and bone age.

In the pediatric cohort, the participants’ responses to kisspeptin-10 could accurately predict those who later progressed through puberty denoted “kisspeptin-responders (LH ≥ 0.8 mIU/mL)” compared to “kisspeptin non-responders (LH ≤ 0.4 mIU/mL)” who did not progress through puberty. Sensitivity and specificity for the kisspeptin-stimulation test were both 100% (95% CI 74%-100%) which predicted outcomes more accurately than previously described basal/stimulated hormonal markers and genetic testing (136). Data from these studies demonstrate the promise of a kisspeptin test of hypothalamic function in the context of delayed puberty and warrant larger studies (Figure 4).

The ability of kisspeptin to directly stimulate hypothalamic GnRH release offers novel insight into the physiology of the hypothalamic GnRH neuronal network. Exogenous kisspeptin was utilized in studies as an *in vivo* interrogator of the GnRH neuronal function in CHH and CDGP. Serum kisspeptin levels can distinguish between different causes of CPP.

CDGP, constitutional delay of growth and puberty; CHH, congenital hypogonadotropic hypogonadism; CPP, central precocious puberty; LH, luteinizing hormone. Figure created with BioRender.com.

#### Precocious puberty

Serum kisspeptin levels were first measured as a potential marker of precocious puberty in 2009 (137). In girls with CPP, serum kisspeptin levels were found to be significantly higher than in age-matched prepubertal controls (14.62 ± 10.2 pmol/l *vs* 8.35 ± 2.98 pmol/l) (137), however there was some overlap between the two groups. A recent systematic review and meta-analysis included 316 CPP patients and 251 controls from 11 studies (138). Consistent with the first study (137), kisspeptin levels were found to be higher in the CPP compared to controls; the bias-corrected standardized mean difference (SMD) was 1.53 (95% CI 0.56-2.51) (138). Subgroup analyses showed a positive correlation between serum kisspeptin and age in the CPP cohort, and an association between serum kisspeptin levels and precocious thelarche (138). However, as noted previously there are overlaps between the two cohorts. Kisspeptin levels could therefore complement current diagnostic tools in precocious puberty (Figure 4).

## 2 Reproduction

Following pubertal transition and attainment of reproductive capacity, maintenance of the reactivated HPG axis function is indispensable for fertility and successful reproduction. Key physiological processes in the menstrual cycle such as folliculogenesis and ovulation are tightly regulated by intricate negative and positive feedback mechanisms in response to sex-steroids (and other signals) with patterns of GnRH and subsequent LH release varying during different phases of the menstrual cycle. During follicular development, pulsatile GnRH secretion is modulated by negative feedback from circulating E2 with LH pulses occurring approximately every hour, whilst during the preovulatory stage, high E2 concentrations exert positive feedback to result in the mid-cycle LH surge and ovulation following which LH pulse frequency progressively lengthens to every 2 to 4hrs during the luteal phase (61).

The activity of kisspeptin neurons varies throughout the menstrual cycle modulated by E2 levels. Whilst both the arcuate KNDy neurons and RP3V kisspeptin neurons express receptors for sex-steroids including estrogen receptor alpha (ERα) (139), progesterone (140) and androgen receptors (141) these two kisspeptin-expressing neuronal populations are associated with disparate functions. During the majority of the follicular and luteal phases, E2 exerts negative feedback and inhibits arcuate KNDy neurons, thus plays a role in the maintenance of pulsatile GnRH secretion. In the late follicular phase, high E2 stimulates RP3V kisspeptin neurons through positive feedback resulting in the GnRH/LH surge responsible for ovulation. This was supported by the differential regulation effects of E2 on RP3V *Kiss1* gene expression (stimulatory) and arcuate *Kiss1* expression (inhibitory) (142–144). Correspondingly, as the GnRH/LH surge, which is essential for ovulation, occurs exclusively in females, RP3V kisspeptin neurons demonstrate marked sexual dimorphism with increased *Kiss1* in females compared to males. During the luteal phase, progesterone from corpora lutea acts to slow pulsatile GnRH and LH secretion. Following exogenous progesterone administration, the ovine *Kiss1* mRNA expression is reduced (145) whilst *Pdyn* mRNA expression is increased (146), thus supporting the potential role of KNDy neurons in mediating this homeostatic-negative feedback to regulate GnRH and LH pulse generation. This putative role was recently investigated using mice with conditional progesterone receptor deletion from KNDy neurons which demonstrated that whilst females have significantly fewer pups, there were no observable effects on estrous cyclicity, LH pulse parameters, or the ability of exogenous progesterone to mediate LH suppression (147). Thus, the loss of progesterone receptor from arcuate KNDy neurons (89% knockout) is insufficient to disrupt negative feedback regulation of GnRH pulses in female mice, suggesting that the small number of remaining KNDy neurons may be sufficient or indeed that other cells may be implicated to regulate GnRH pulse generation (147).

In premenopausal women, the gonadotropin response to exogenous kisspeptin is dependent on the endogenous sex-steroid milieu. During most phases of the cycle, the LH response to kisspeptin is modest and less than that in eugonadal men (27,44,148–150). In healthy adult men, a 90 minute infusion of kisspeptin-54 (4pmol/kg·min) led to mean stimulated LH of 10.8 ± 1.5 IU/L *vs* 4.2 ± 0.5 IU/L following saline control (27). Similarly, an infusion of kisspeptin-10 (4μg/kg·hr) led to a robust rise in LH from a mean of 5.4 ± 0.7 to 20.8 ± 4.9 IU/L (44). In females, a subcutaneous bolus of kisspeptin-54 increased plasma LH compared with saline in all phases of the cycle, however the greatest LH rise was seen in the preovulatory and least in the follicular phase (mean increase in LH over baseline in follicular phase: 0.12 ± 0.17 IU/L; preovulatory phase: 20.64 ± 2.91 IU/L and luteal phase: 2.17 ± 0.79 IU/L) (29). This differential response is also evident following kisspeptin-10, where during the follicular phase, no rise in serum gonadotropins was observed, whereas in contrast during the preovulatory phase, serum LH and FSH were elevated after an intravenous bolus of kisspeptin-10 (10 nmol/kg) with a mean LH area-under-the-curve (AUC) increase of 30.3 ± 7.7 h·IU/L) (149). The incremental LH response to kisspeptin solely in the late follicular/preovulatory phase of the menstrual cycle evidences the role for kisspeptin in the preovulatory positive estrogenic drive to GnRH/LH secretion (29,31,45,149,151,152).

### Kisspeptin and NKB in the physiology of ovulation

In ovariectomized mice, during an LH surge induced with exogenous gonadal steroids, ~30% of RP3V kisspeptin neurons expressed c-Fos compared to none in non-surging controls (153). Notably, there was a strong correlation between the percentage of c-Fos-positive kisspeptin neurons and the percentage of c-Fos-positive GnRH neurons (153). The LH surge was absent in *Kiss1* (154) and *Kiss1r* knockout rodent models with low functional c-Fos-GnRH activity (154). Moreover, continuous intracerebroventricular injection of a kisspeptin receptor antagonist prevented the preovulatory LH surge in adult cycling female rats in the proestrus phase and in the sheep (83,155). Thus, kisspeptin signaling is essential for GnRH neuronal activation that initiates ovulation.

To investigate the role of kisspeptin in the physiological positive estrogen feedback that induces the ovulatory LH surge in women (156), exogenous estrogen was administered to achieve sufficient plasma E2 levels to induce ovulation. Treatment with exogenous estrogen for 32hrs increased serum E2 and serum LH at 48hrs, which continued to be elevated at 72hrs. Kisspeptin-10 infusion was able to stimulate LH secretion with the degree of LH rise proportional to serum E2 concentrations at the start of the infusion (156). Congruous with preclinical data, kisspeptin appears to be a key component of the preovulatory LH surge through direct GnRH stimulation.

The potential role of locally expressed kisspeptin, NKB and their cognate receptors in the ovaries is increasingly recognized. Within the ovaries NKB/NK3R and kisspeptin/KISS1R is expressed in the uterus, ovary, oviduct (157) and within ovarian granulosa cells (158). Studies using *Kiss1r* haplo-insufficient mice model led to premature ovarian insufficiency, progressive loss of developing follicles despite preserved gonadotropin levels thus substantiating the importance of direct kisspeptin signaling in the ovaries (159). *In vitro* applications of kisspeptin and NKB in follicular cells induced expression of steroidogenic enzymes (160), local growth factors to regulate ovarian cells’ viability, proliferation, apoptosis, and hormone release (161). The NKB/NK3R and kisspeptin/KISS1R system may therefore be important in the autocrine/paracrine regulation of follicular development, oocyte maturation, ovulation and ovarian steroidogenesis (162). In women with PCOS, expression studies demonstrated upregulation of *KISS1* and *KISS1R* (163) and downregulation of *NK3R* mRNA (160) in granulosa cells compared to eumenorrheic controls. Further understanding of the physiology of NKB/NK3R and kisspeptin/KISS1R systems in the ovary may therefore advance our knowledge of the pathogenesis of ovulatory and reproductive disorders.

### Ovulatory disorders

Ovulatory disorders are common causes of oligo/amenorrhea and subfertility. The International Federation of Gynecology and Obstetrics (FIGO) guidelines primarily classifies ovulatory disorders into four groups: Type I: Hypothalamic; Type II: Pituitary; Type III: Ovarian and Type IV: polycystic ovary syndrome (PCOS) (164). With the exception of Type III: Ovarian, the other causes of ovulatory disorders involve the neuroendocrine control of GnRH function.

Functional hypothalamic amenorrhea (FHA) is one of the most common causes of amenorrhea and ovulatory dysfunction being present in 53% and 72% of primary and secondary amenorrhea, respectively (165). FHA is characterized by low body-weight, excessive exercise, and stress, on a background of genetic susceptibility. The resultant reduced energy availability associated with hypoleptinemia, results in reduced GnRH neuronal function and a top-down disruption of the HPG axis with detrimental impact on fertility, bone, and cardiovascular health (166).

Caloric restriction models are frequently employed to evaluate the impact of low body weight and hypoleptinemia on reproductive phenotypes. Under chronic undernutrition, gonadally intact female mice experienced rapid weight loss, cessation of estrus cyclicity, a significant decrease in uterine/ovarian weight and number of corpora lutea. *Kiss1* mRNA expression in the arcuate and RP3V nucleus were reduced resulting in marked suppression of pulsatile LH secretion and E2-induced LH surge (167). As discussed above, the effect of low circulating leptin levels on the reproductive axis in FHA is likely mediated through intermediary neurons including AgRP/NPY and POMC/CART neurons that abundantly express LepR as well as insulin receptor and growth hormone secretagogue receptor (168–171), which are cognate receptors for leptin, insulin, and ghrelin, respectively. These metabolic hormones are major endocrine signals of energy reserves. In addition to their key roles as gatekeepers of pubertal development these neurons also integrate and finetune the metabolic hormones’ permissive signals for healthy cyclicity and ovulation. For example, chemogenetic activation of AgRP neurons disrupts rodent estrus cyclicity, increases duration of diestrus phase and time to conception (107) as well as decreasing LH secretion post-gonadectomy (172), mimicking the FHA phenotype.

PCOS affects 2-13% of reproductive age women and is traditionally diagnosed based on the presence of 2 of the following 3 criteria: (i) menstrual irregularity, (ii) hyperandrogenism (clinical or biochemical) and (iii) polycystic ovarian morphology (173). PCOS is characterized by elevated LH pulse frequency, androgen excess, which causes impaired suppression of GnRH secretion in response to sex-steroid induced negative feedback (174). The raised LH to FSH ratio (due to increased GnRH pulsatility) gives rise to the reproductive phenotypes through stimulation of androgen secretion from thecal cells and preovulatory follicle arrest. In PCOS, impaired negative feedback to E2 and progesterone indicates neuroendocrine disruption which impair the ability of steroid hormones to restrain GnRH/LH pulse frequency (175). Indeed, antagonizing the androgen receptor could restore sensitivity to sex-steroid mediated feedback (176). The lack of androgen receptors on GnRH neurons implies involvement of afferent intermediary neurons such as kisspeptin-expressing neurons. A prenatal androgen-treated mouse model of PCOS demonstrated elevated androgen receptor gene expression in KNDy cells whilst significant reductions in progesterone receptor and dynorphin gene expression were observed suggesting impaired negative feedback to KNDy cells (141). Furthermore, synaptic inputs from hypothalamic regions sensitive to sex-steroids to KNDy neurons were reduced (141).

**Figure 5:**
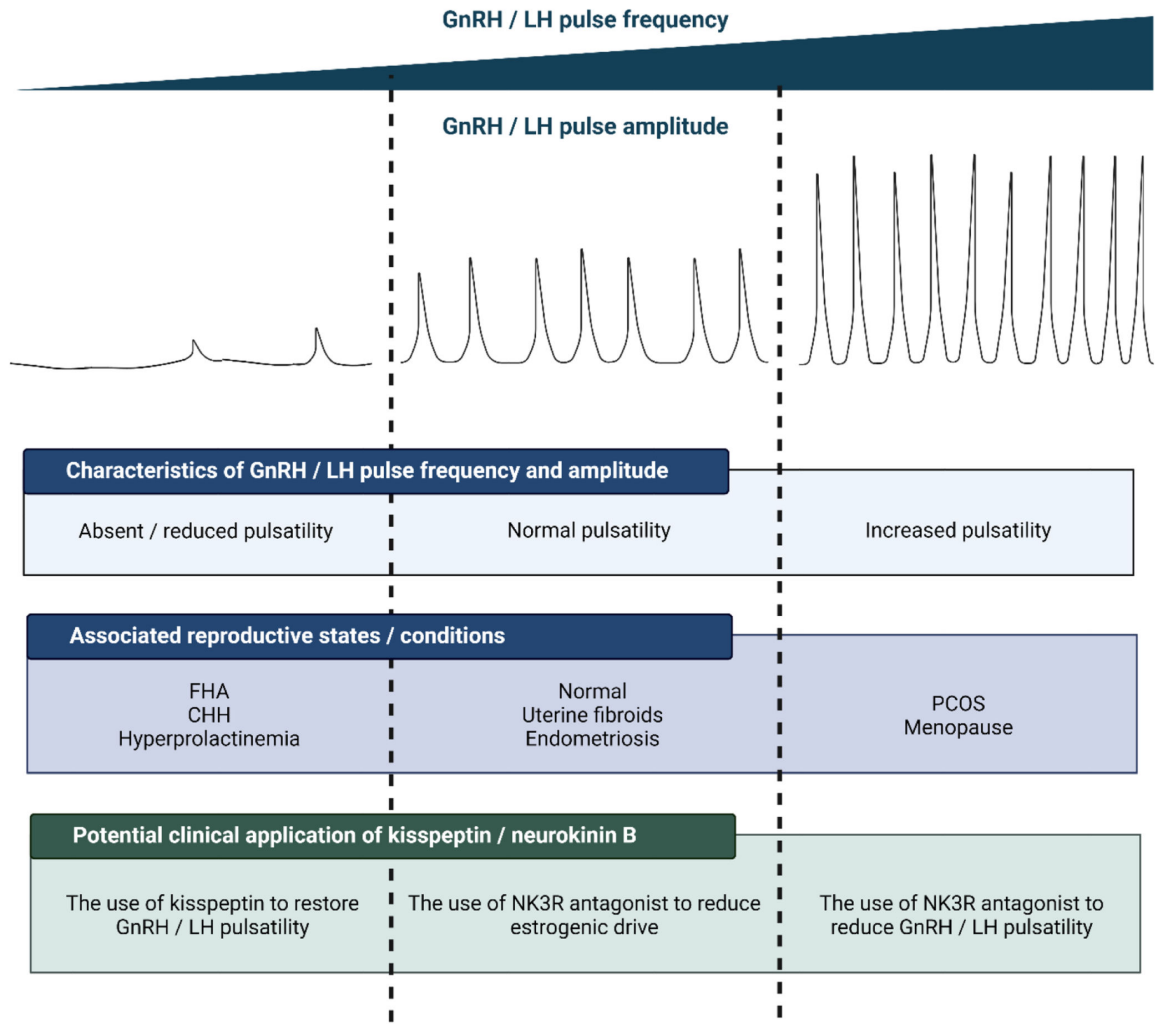
Therapeutic potential of kisspeptin and neurokinin B in female reproductive disorders. Activation of hypothalamic kisspeptin neurons directly stimulates GnRH release and regulates reproductive hormone secretion. Absent or reduced GnRH and LH pulses are observed in FHA, CHH, and hyperprolactinemia. In functional causes (FHA or hyperprolactinemia), LH pulses can potentially be restored using exogenous kisspeptin. While GnRH/LH pulsatility remains unaltered in patients with endometriosis/uterine fibroids, patients with PCOS have high pulsatility. Considering NKB antagonism partially suppresses (but does not abolish) the reproductive endocrine axis, NK3R antagonists have therapeutic potential for endometriosis / uterine fibroids (by reducing E2) and PCOS (by reducing androgens and heightened GnRH pulsatility).

As the pathogenesis underlying both FHA and PCOS involve neuroendocrine dysregulation of GnRH pulsatility and ovulation; the kisspeptin/NKB system, with its key physiological role as the GnRH pulse generator, has therefore emerged as a prime neuronal population to integrate internal homeostatic factors to fine-tune the final neuronal output (Figure 5). Considerable research in the clinical application of kisspeptin and NKB has therefore been undertaken as detailed below.

CHH, congenital hypogonadotropic hypogonadism; FHA, functional hypothalamic amenorrhea; GnRH, gonadotropin-releasing hormone; LH, luteinizing hormone; NKB, neurokinin B; NK3R, neurokinin 3 receptor; PCOS, polycystic ovary syndrome. Figure created with BioRender.com.

### Potential diagnostic and therapeutic utilities of kisspeptin in ovulatory disorders

#### Kisspeptin for the diagnosis of ovulatory disorders

Assessment of circulating kisspeptin levels may have diagnostic utility and be used to differentiate ovulatory disorders. Circulating kisspeptin levels are lower in women with FHA, particularly in those with a reduced LH, compared to healthy women on days 11-13 of the menstrual cycle (177,178). In keeping with this, kisspeptin levels also negatively correlated with physical activity (179). Conversely, a meta-analysis of 12 studies reported that circulating kisspeptin levels were higher in women with PCOS than in healthy controls with a pooled AUC of 0.835 and pooled odds ratio (OR) of 13.71 when differentiating women with PCOS from BMI-matched controls (180). A further case-control study also demonstrated that PCOS was associated with increased kisspeptin levels (181). However, at present the challenges of accurately detecting low serum kisspeptin levels using current assays limit its potential clinical use.

The ability of kisspeptin to directly stimulate GnRH secretion enables its potential use as a diagnostic test to interrogate hypothalamic GnRH neuronal functioning. Men with CHH demonstrated a reduced LH and FSH response to an intravenous bolus of kisspeptin-54 compared to eugonadal men (135). A subcutaneous bolus of kisspeptin-54 stimulates a greater rise in LH in women with FHA compared to eumenorrheic controls (182). The kisspeptin receptor agonist, MVT-602, demonstrated a similar degree of LH increase in both healthy women and those with PCOS, but an augmented and expedited rise in women with FHA (152). These differential responses offer the possibility of kisspeptin being utilized as a diagnostic test to differentiate between ovulatory disorders pending further studies.

#### Kisspeptin as potential treatment for ovulatory disorders

##### FHA

Kisspeptin could have therapeutic utility for functional hypogonadal disorders with hypothalamic dysfunction such as FHA given its ability to directly stimulate the hypothalamus. FHA is characterized by a loss of the physiological pulsatile release pattern of GnRH and LH subsequently leading to reduced folliculogenesis, low E2 production and anovulation (183). The recommended first-line treatment for FHA following lifestyle modification is pulsatile GnRH pump therapy to replace the lack of pulsatile GnRH release in FHA, but limited availability of these pumps precludes its clinical utility (31,166,184). Estrogen supplementation provides symptomatic relief and benefits to bone mineral density (BMD) but does not address fertility issues (31,184). Furthermore, the use of clomiphene citrate, a selective estrogen receptor modulator, which reduces the E2 mediated negative feedback to increase endogenous gonadotropin secretion, often has limited effect due to the inherently hypoestrogenic state associated with FHA (31,184). Recombinant leptin treatment is also not desirable for use in women with FHA as it can cause weight loss (99,166). Kisspeptin-54 administration can increase LH pulsatility in women with FHA even when administered in a non-pulsatile manner with a greater LH rise than in healthy women (31,182). Indeed, the response to kisspeptin is increased in FHA, which could be by a compensatory increase in the kisspeptin receptor, as observed in caloric-restricted rodent models (102). However, chronic administration protocols are required to facilitate its therapeutic use in FHA.

Chronic administration of twice daily kisspeptin-54 in FHA resulted in tachyphylaxis, with the LH response to kisspeptin-54 being significantly diminished by day 14 of administration (182). This phenomenon is hypothesized to be secondary to kisspeptin receptor desensitization, with response to GnRH maintained after kisspeptin treatment, demonstrating intact pituitary response (182). Tachyphylaxis with chronic kisspeptin administration has been demonstrated across several species and is more likely to occur with frequent and high dose administration (185). Tachyphylaxis also appears to be influenced by sensitivity to kisspeptin, with the same dosing schedule used in women with FHA, not inducing tachyphylaxis during the follicular phase of healthy women when they are less sensitive to kisspeptin (29,149). Extending the dosing interval of kisspeptin-54 to twice weekly enabled persistent LH stimulation over 8 weeks of treatment, although menstrual cyclicity was not restored (186). Thus, an intermediate dosing regimen between twice daily and twice weekly could be required for this indication.

Continuous infusion of kisspeptin could provide an alternative method for administration for chronic stimulation in women with FHA. Administration of kisspeptin-54 to women with FHA via continuous intravenous infusion over 8 hours led to a dose-dependent increase in mean LH and FSH levels, with an intermediate dose having the greatest effect on LH pulsatility (187). Thus, continuous administration of kisspeptin at lower doses could maintain LH pulsatility but avoid causing desensitization. However, studies utilising chronic continuous kisspeptin administration to identify the optimal dose to achieve stimulation persistently in this population are still required.

Kisspeptin receptor agonists also provide a potential therapeutic option in FHA. MVT-602 (previously known as TAK-448) was developed through modification of kisspeptin-10 to form a nonapeptide that has increased stability, water solubility, and potency (152). MVT-602 has a similar pharmacokinetic profile to kisspeptin-54, with a half-life after subcutaneous injection of 1.5hrs, however in healthy women in the follicular phase it caused a more sustained rise in LH (152). When administered to women with FHA, MVT-602 resulted in an advanced LH response compared with healthy women with a greater rise in FSH than in the follicular phase of healthy women (152). MVT-602 administration has exhibited tachyphylaxis when given as a high-dose subcutaneous infusion in men (188). However, the response to a single subcutaneous bolus of MVT-602 in women with FHA was sustained for 48hrs and could facilitate infrequent low-dose bolus chronic administration protocols for FHA treatment to mitigate against tachyphylaxis (152). Further studies are needed to evaluate whether low dose intermittent administration of MVT-602 can achieve persistent stimulation and therefore be appropriate for a chronic administration protocol, especially given its increased potency (152).

##### Hyperprolactinemia

Kisspeptin could also have a therapeutic role in other causes of functional hypogonadism due to hypothalamic dysfunction such as hyperprolactinemia. Hyperprolactinemia results in hypogonadotropic hypogonadism through suppression of kisspeptin afferents to GnRH neurons resulting in reduced GnRH/LH pulse frequency and amplitude (189–191). An intravenous infusion of kisspeptin-10 for 12hrs significantly increased LH, FSH and E2 levels, and increased LH pulsatility in women with chronic hyperprolactinemia-induced hypogonadotropic amenorrhea with cabergoline-resistant microprolactinomas (190). Likewise, repeated intravenous bolus administration of kisspeptin-10 also increased LH levels in women with hyperprolactinemia (189). This may offer a treatment option to restore ovarian function in cases of hyperprolactinemia where dopamine agonists are ineffective or not tolerated and pituitary surgery is not an option. However, kisspeptin administration would not be expected to affect prolactinoma size, and safety of restoring ovulation should be carefully considered by the multidisciplinary team.

##### PCOS

Current treatment strategies for PCOS target specific symptoms rather than the underlying pathophysiological process. PCOS is characterized by an abnormally increased GnRH pulse frequency, which is reflected by LH predominant secretion with arrested follicle development resulting from the relative FSH deficiency (192). This increased LH promotes ovarian hyperandrogenism, which in turn reduces sex-steroid negative feedback to hypothalamic kisspeptin neurons, to further drive increased LH secretion, establishing a vicious cycle. When kisspeptin-10 was administered to women with PCOS, the response was LH predominant, demonstrating a positive association with pre-treatment E2 concentration but with little FSH response (193). However, following pre-treatment for 1 week with an oral neurokinin-3 receptor antagonist an FSH response was observed following kisspeptin-10 administration with a maintained LH response. Neurokinin-3 receptor antagonist administration reduced FSH and LH secretion and LH pulse frequency, which could facilitate the differential response to kisspeptin-10 observed and help restore folliculogenesis (193). The kisspeptin receptor agonist MVT-602 may also provide a promising alternative option. When administered to women with PCOS, MVT-602 causes both an LH and FSH rise to a similar degree as healthy women in the follicular phase, and could be used to trigger ovulation during ovulation induction cycles pending further studies (152).

The efficacy of chronic administration of kisspeptin to oligo/anovulatory women with PCOS has been investigated. Twice daily subcutaneous kisspeptin-54 for 3 weeks resulted in an overall small rise in LH, but no rise in FSH (194). Furthermore, only 2 women with oligo/anovulatory PCOS subsequently ovulated. A similar finding was demonstrated using rodent models of PCOS where in anovulatory rats with neonatal androgen exposure, a bolus of kisspeptin-54 resulted in marked LH and FSH responses and rescued ovulation. However, in post-weaning androgenized rats with persistently raised androgen levels, the LH response to kisspeptin-54 was blunted and there was no resulting ovulation (194). This study evidences the variability of endocrine profile in women with PCOS and how it will likely influence the subsequent response to kisspeptin. Therefore, an individualized approach is required when approaching the management of patients with PCOS.

### NKB as potential treatment for ovulatory disorders

NKB antagonism has also been an area of interest in PCOS treatment. Women with PCOS with inactivating variants of genes encoding for NKB or its receptor, have low baseline LH and LH pulsatility (89,90). However, mouse models with absent NKB signaling can still generate LH pulses (195). Thus, antagonism of NKB action could target the pathophysiological process underlying the increased LH secretion and hyperandrogenism observed in PCOS through normalization of GnRH pulsatility. Women with PCOS who received the oral neurokinin-3 receptor antagonist MLE4901 at 80mg/day had a 52% baseline-adjusted reduction in the AUC of LH, a 79% reduction in basal LH secretion, and an LH pulse decrease of 3.6 pulses/8hrs after 7 days of treatment compared to placebo (196). Furthermore, total testosterone and free testosterone levels were reduced by 29% and 19% respectively (196). A subsequent study administered MLE4901 at 40mg twice daily to women with PCOS and showed a reduction in LH secretion (from 6.5 to 4.0 IU/L), as well as in LH pulse frequency and FSH levels compared to placebo (193). Another neurokinin-3 receptor antagonist, Fezolinetant, has also been trialed in women with PCOS at dose of 60mg and 180mg for 12 weeks, causing reduced LH, FSH, total testosterone levels and LH:FSH ratio in a dose-dependent manner (197). No changes were observed in E2 and progesterone levels, endometrial thickness, follicle development, or menstrual cycle irregularity in this study.

### The role of kisspeptin in *in vitro* fertilization (IVF)

Subfertility affects 1 in 6 couples and is defined as the inability to conceive following 12 months of regular unprotected sexual intercourse (198). *In vitro* fertilization (IVF) is one of the main treatment options for infertility, with a 4-5% annual increase per year in the number of IVF cycles undertaken in the UK (199). During IVF, supraphysiological doses of FSH are used to induce multi-follicular growth in the ovaries. Premature ovulation is prevented by administration of competitive GnRH antagonist or by chronic administration of GnRH agonist (short *vs* long protocol respectively). Once the follicles reach a size threshold of 17-18mm, LH receptor agonism is provided to induce oocyte maturation (resumption of the first meiotic division and luteinization of granulosa cells) and ovulation. Human chorionic gonadotropin (hCG) or GnRH agonists are usually used in current clinical practice to provide this LH receptor agonism (200).

**Figure 6:**
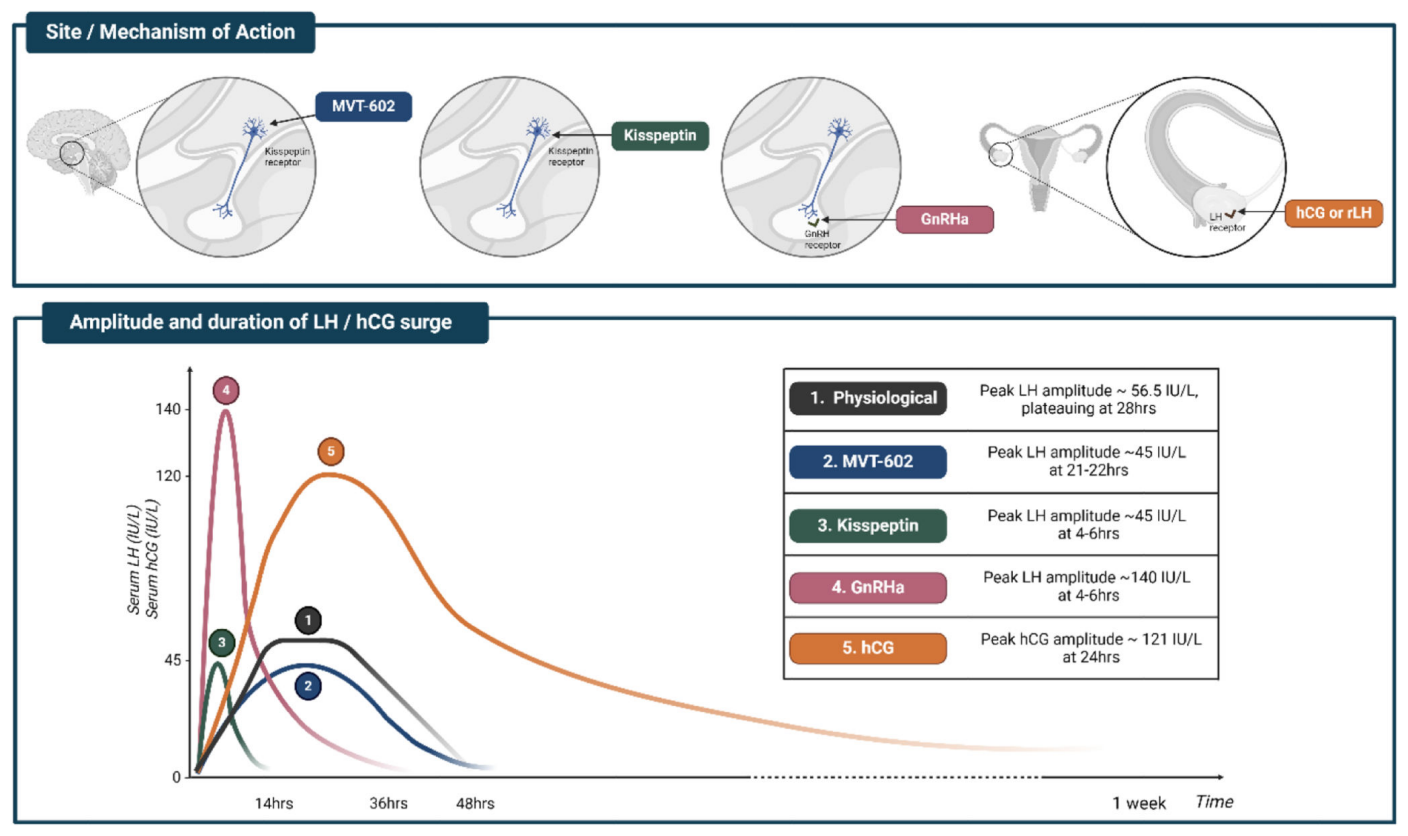
Site of action of oocyte maturation triggers and the resultant serum LH or hCG response compared to physiological LH amplitude. The physiological LH surge during a natural cycle has a mean duration of 48hrs. This is divided into three phases; the first short ascending phase lasting 14hrs, the second peak plateau phase reaching an average amplitude of 56.5 IU/L lasting 14hrs and lastly a long descending phase of 20hrs.

The physiological LH surge during the natural menstrual cycle has a mean duration of 48hrs with three phases; firstly a short ascending phase lasting 14hrs, secondly a peak plateau phase reaching an average amplitude of 56.5 IU/L with a standard deviation of 23.4 (range 25-114 IU/L) (201) lasting 14hrs, and lastly a long descending phase of 20hrs (202). Kisspeptin has been shown to induce an amplitude of LH rise more in keeping with that of the physiological mid-cycle LH surge compared to either hCG or GnRH agonists (203–205). The peak LH level following a kisspeptin-54 trigger was 41.4 IU/L at 4hrs post-administration (206,207). The kisspeptin receptor agonist, MVT-602, has recently been characterized in healthy women and in women with reproductive disorders (152). In the healthy follicular phase, the amplitude of LH rise was similar to that after kisspeptin-54, however the duration was markedly prolonged compared to kisspeptin-54 (time of peak LH: MVT-602 21-22hrs *vs* kisspeptin-54 4.7hrs) leading to a more than four-fold increase in the area under the curve of the LH exposure (152). In a minimal stimulation cycle, the mean increase in LH from baseline was 82.4 IU/L at ~25hrs following administration of 3 μg MVT-602 and remained elevated to >15 IU/L for 33hrs (208). Thus, MVT-602 appears to induce an LH profile that is most similar to that of the endogenous LH surge. In contrast, GnRH agonist trigger results in a supraphysiological peak LH level of 140.4 IU/L at 4hrs post-administration, and hCG levels peak ~24hrs post administration at 121.0 IU/L (Figure 6) (207). Therefore, kisspeptin appears to be a promising alternative agent providing a more physiological LH profile for induction of oocyte maturation in IVF protocols.

Kisspeptin/MVT-602 acts at the level of the hypothalamus to stimulate kisspeptin receptors on GnRH neurons leading to GnRH release. Kisspeptin induces a peak LH rise of ~45 IU/L at ~5hrs, returning to pre-trigger levels at 12-14hrs. MVT-602 induces a peak rise in LH of similar amplitude to that of kisspeptin-54, however the duration of the LH rise was markedly prolonged, with peak LH occurring at ~21-22hrs. GnRHa acts at the level of the anterior pituitary gonadotrophs to stimulate endogenous LH and FSH secretion. GnRHa induces a peak LH level of 140.4 IU/L at 4-6hrs after administration.

hCG and rLH act at the level of the ovary, directly on LH receptors. A subcutaneous bolus of hCG results in a peak hCG level of 121.0 IU/L at 24hrs after administration.

GnRHa, gonadotropin-releasing hormone agonist; hCG, human chorionic gonadotropin; LH, luteinizing hormone; rLH, recombinant luteinizing hormone. Figure created with BioRender.com.

Indeed, kisspeptin-54 was used as a trigger for oocyte maturation in a proof-of-concept study in 2014 (203). A single subcutaneous bolus injection of kisspeptin-54 (dose-range between 1.6 and 12.8 nmol/kg) in 53 women with infertility undergoing a GnRH antagonist co-treated IVF cycle (203). This resulted in the successful retrieval of at least one mature oocyte in 51 of 53 women (96.2%), one embryo for implantation in 49 of 53 women (92.5%), and the birth of 12 healthy babies (203).

A major complication of established protocols using hCG as the oocyte maturation trigger is the risk of ovarian hyperstimulation syndrome (OHSS), which can be life-threatening with severe forms occurring in 2-6% of IVF cycles (209–211). This can be attributed to the prolonged duration of action of exogenously administered hCG lasting ~10 days (212). OHSS occurs due to excessive ovarian stimulation causing release of vascular endothelial growth factor (VEGF) and increased vascular permeability, resulting in fluid shifts from the intravascular to the third space compartments (209). This can lead to ascites, pleural effusions, and renal failure (209). Current strategies employed in IVF practice to reduce OHSS risk include the use of GnRH antagonist protocol to prevent premature ovulation during ovarian stimulation and the use of a GnRH agonist to trigger final oocyte maturation, however these techniques can have unwanted effects on efficacy, such as lengthening the time to pregnancy, or increasing the risk of late pregnancy complications such as pre-eclampsia (209).

Kisspeptin-54 was investigated as an oocyte maturation trigger in women at high risk of OHSS defined as total antral follicle count of >23 or serum AMH level ≥40 pmol/L (204). In 60 women at high risk of OHSS, kisspeptin safely induced oocyte maturation with no cases of moderate, severe, or critical OHSS (204). A single subcutaneous bolus of kisspeptin-54 (dose range 3.2-12.8 nmol/kg) resulted in oocyte maturation in 95% of women and this rate increased in a dose-dependent manner: 53% at 3.2 nmol/kg, 86% at 6.4 nmol/kg and 9.6 nmol/kg, and 121% at 12.8 nmol/kg (204). Embryo formation occurred in 90% of women with resulting biochemical pregnancy, clinical pregnancy, and live birth rates per transfer of 63%, 53%, and 45% respectively (204). In a single-center retrospective study, the risk of OHSS was markedly increased with hCG (OR 33.6; CI, 12.6-89.5) or a GnRH agonist (OR 3.6; CI 1.8-7.1) than kisspeptin-54 (213).

Even though the amplitude of LH exposure induced by kisspeptin-54 (~45 IU/L) is similar to that of the physiological midcycle LH surge (56.5 IU/L); the duration of LH exposure induced by kisspeptin-54 is shorter than the triphasic physiological LH surge. To determine if the duration of LH-exposure impacts on IVF outcomes, 62 women at high risk of OHSS were randomized to receive either one or two doses of kisspeptin-54 to trigger oocyte maturation (205). This second dose of kisspeptin-54 induced further LH secretion at 4hrs after the second injection, providing a ‘rescue’ response in those who had a lower LH rise following the first kisspeptin-54 dose (205). This prolonged LH-exposure improved the number of women achieving at least 60% oocyte yield (71% *vs* 45%), implantation rates (37% *vs* 23%), and live birth rates (39% *vs* 19%) compared to those receiving a single dose (205). Importantly, this prolonged LH exposure did not cause an increase in clinically significant OHSS in these high-risk women (205). Besides its shorter duration of action, kisspeptin also appears to have direct action on ovarian kisspeptin receptors to suppress VEGF release which may contribute to the reduction in the incidence of OHSS (204).

Live birth rate per embryo transfer following all kisspeptin doses tested appears to be at least comparable to currently used triggers: 32% (51/160) (200) and up to 45% (23/51) (204) in high responders with contemporaneous live birth rate per transfer in women <35 years treated with fresh embryo transfer being 32.8% (214). Thus, a prospective comparison of the safety and efficacy of kisspeptin against current agents is warranted.

### The role of NKB in gynecological disorders including endometriosis and uterine fibroids

Endometriosis and uterine fibroids are common disorders affecting women of reproductive age and are leading causes of pelvic pain, abnormal uterine bleeding, and subfertility (215,216). Early age of menarche and short menstrual length are both associated with development of uterine fibroids and endometriosis (215,216) consistent with prolonged endometrial and myometrial exposure to estrogen being an important factor in the pathogenesis of these disorders (215,216). Lowering E2 levels to between 110 to 184 pmol/L has been recommended as being effective in reducing the symptoms of uterine fibroids and endometriosis (217,218). GnRH modulators are able to suppress estrogen levels by shutting down the HPG axis top-down and are currently validated treatment for both conditions on this basis (219,220). However, these agents typically induce lower E2 and require add back sex-steroid replacement to avoid the associated adverse effects (221,222). NKB receptor antagonists could potentially be dosed to reduce E2 levels into the recommended therapeutic range sufficient to moderate endometriosis and uterine fibroid development without the adverse effects of undetectable E2 levels.

Several NKB receptor antagonists have demonstrated the ability to reduce LH secretion whilst preserving FSH secretion. The NK3R antagonist MLE4901 (also known as AZD4901, formerly AZD2624) reduced basal LH secretion in healthy women without affecting the LH pulse frequency during the early-mid follicular phase and delayed the LH surge by 7 days, whilst FSH secretion was unaffected (223). MLE4901 has also been shown to reduce E2 secretion, endometrial thickness and folliculogenesis during the follicular phase (224). Another NK3R antagonist, Fezolinetant (ESN364), also led to a dose-dependent reduction in serum LH and delayed the LH surge in healthy women, with no significant effect on FSH (225). Fezolinetant subsequently caused a dose-dependent delay to the rise in serum E2 levels during the follicular phase, but the serum E2 trough level remained above the 110 pmol/L, a threshold considered significant for inducing menopause-like symptoms (225). The dual neurokinin 1, 3 receptor antagonist Elinzanetant induced a trend towards a dose-dependent reduction in serum LH when administered once daily to healthy women for 21 days with no safety concerns or issues with tolerance (226). Elinzanetant also caused a dose-dependent reduction in serum E2 and progesterone levels, with 120mg/day causing a median reduction in serum E2 to 141 pmol/L, which is within the recommended range to effectively treat uterine disorders (226). Furthermore, the 120mg Elinzanetant dose increased the menstrual cycle length by a median of 7 days. This suggests that NKB antagonism may provide a novel therapeutic approach, and further study is required to evaluate its use in uterine disorders.

## 3 Reproductive Behavior

Efficient reproductive strategies are crucial cornerstones for the continuity of survival of any species. The timing of reproductive activity in many species results from the integration of internal physiological processes and behavioral cues, aligning precisely with the optimal window for fertilization. The evolution of sexual desire, deriving pleasure, and arousal from these experiences serves as a compelling force for many species, including humans, urging individuals to engage in sexual encounters and, consequently, frequently facilitating reproduction. Indeed, this orchestration of reproductive function and sexual behavior appears to be mediated by common hormonal factors, among which estrogen, testosterone and kisspeptin prominently feature with the role of NKB in these processes yet to be fully explored.

### The physiology of kisspeptin in reproductive behavior

#### Anatomical insights

In addition to its reproductive roles mediated largely by hypothalamic kisspeptin neurons, kisspeptin and its cognate receptor are also expressed in extra-hypothalamic regions, particularly within the mammalian limbic and paralimbic system, which consists of areas that are implicated in mood, behavior, sexual desire, and function (227).

In rodents, *Kiss1r* mRNA was detected in the amygdala, thalamus (caudate nucleus, globus pallidus, putamen), hippocampus, para-hippocampal gyrus, medial and superior frontal gyrus, and striatum of rats (12,13,15). *Kiss1r* mRNA is similarly expressed in the limbic and paralimbic areas of mice, with highest expression in the dentate gyrus of the hippocampus (228). In both rats and mice, there is a well-established kisspeptin neuronal population in the posterodorsal medial amygdala (mePD), a neural locus in the limbic brain involved in the regulation of sexual behaviors including investigation and attraction towards opposite sex-conspecifics, as well as emotion, such as fear and anxiety. This is indeed a critical area in the modulation of sexual behavior through both sex-steroids and kisspeptin (229).

Similarly in humans, extrahypothalamic expression of *KISS1R* mRNA and protein has been detected by reverse transcription polymerase chain reaction (rtPCR) in the amygdala, caudate nucleus, cingulate gyrus, globus pallidus, hippocampus, medial frontal gyrus, nucleus accumbens, para-hippocampal gyrus, putamen, striatum, substantia nigra, superior frontal gyrus and thalamus (14,15). Kisspeptin expression is also present throughout the aforementioned areas in both rodents and humans, albeit at lower levels (14,230).

#### Sexual mate preference in non-human species

The initiating step to rodent reproduction is detection, recognition, and selection of potential reproductive partner. Sexually active male rodents pursue female conspecifics, while females in estrous phase attempt to engage with males (231). This behavior, recognized as ‘sexual mate preference’, depends partly on the recognition and utilization of various olfactory and acoustic stimuli to maximize their chances of reproduction and species propagation.

Olfactory mediated sexual behavior relies on the detection of pheromones from conspecific of the opposite sex (232). In rodents, pheromones are detected and processed by a highly specialized neural circuit, within the accessory olfactory system, initiating in the vomeronasal organ (VNO) in the nasal septum (233). Wild-type male mice exhibit a significant olfactory preference for female stimuli by spending over 70% of their investigatory time with females. However, *Kiss1r* knockout males displayed no preference for either sex, and allocated equal investigatory time to both males and females irrespective of sex-steroid replacement/milieu (233). Notably, these findings persist, even though *Kiss1r* knockout male mice maintain normosmia, as assessed through a ‘hidden cookie test’, hence demonstrating that the defect was not in distal olfaction (233). Importantly, the olfactory partner preference of *Kiss1r* knockout males remained unaltered by replenishing physiological testosterone levels with treatment, thereby attributing the observations to the absence of intact kisspeptin receptor, rather than any sex-steroid confounders (233).

Kisspeptin’s regulatory role in olfaction-induced sexual partner preference in male mice, appears to be site-specific, with the posterodorsal medial amygdala (MePD) particularly implicated (234). In male mandarin voles, opposite sex pheromonal cues conveyed via the accessory olfactory bulb (AOB) induce c-Fos activation in the MePD kisspeptin neurons, thus suggesting a reciprocal synaptic innervation between the two (235). Similarly, in adult male rats, kisspeptin neuronal fibers in the mitral cell layer of the AOB connect bi-directionally with kisspeptin neuronal fibers in the medial amygdala (MeA), which in turn, make connections with the preoptic area of the hypothalamus (236).Therefore, kisspeptin has a dual role of integrating olfactory pheromonal cues in sexual behavior centers and the HPG axis.

Chemogenetic stimulation of MePD *Kiss1* neurons in male mice presented with a choice between an estrous female and a male, led them to spend twice as long investigating estrus females compared to controls, thereby confirming that kisspeptin activation is implicated in the enhancement of sexual partner preference (234). Furthermore, when wild-type male mice are exposed to female olfactory stimuli, there is a two-fold rise in the number of c-Fos positive MePD kisspeptin neurons, accompanied by a concomitant rise in LH release (237).

Interestingly, the total time spent investigating both conspecifics was also significantly greater suggesting a potential increase in sociability (234). In a comprehensive investigation into sexual motivation, three groups of adult male rats were subjected to appetitive behavioral testing, which focused on measures, such as the frequency of attempts to approach the female and the latency period preceding these attempts, in the presence of an estrous female. Each group received either intranasal GnRH analogue (buserelin), intranasal kisspeptin-10 or intraperitoneal kisspeptin-10 (238). GnRH analogue administration resulted in a three-fold increase in testosterone levels but had no effects on the number of attempts to approach the estrous female, nor influenced the latency period between the attempts. Conversely, intraperitoneal kisspeptin-10 led to modest rise in testosterone levels, while also significantly increasing the number of attempts and decreasing the latency time. Intranasal kisspeptin-10 did not affect testosterone levels, but akin to intraperitoneal kisspeptin-10, led to a significant increase in the number of attempts and a decrease in latency time. This study highlights the testosterone-independent effects of kisspeptin in enhancing sexual motivation in male rodents (238).

When female mice are exposed to male odors, such as male urine or soiled bedding, the RP3V kisspeptin neurons are specifically activated (239) via the VNO (240). Conversely RP3V *Kiss1* activation does not occur when wild type female mice are exposed to same-sex (female) pheromones (239). When ovariectomized female rats are presented with male (but not female) odors, they exhibit increased kisspeptin activity in the RP3V, as well as an augmented LH surge (241). *Kiss1r* knockout female mice, despite intact olfaction, displayed no olfactory preference for either sex, irrespective of their sex-steroid milieu (233). When *Kiss1* expressing RP3V cells are ablated in ovariectomized female mice on adequate estrogen and progesterone replacement (OVX+E+P), they no longer display male-directed preference (240). Notably, male-directed preference is restored on administration of subcutaneous kisspeptin-10 (240). Taken together, these data suggest that RP3V kisspeptin neurons are an essential component of the neural circuits downstream of the VNO mediating olfactory-driven mate preference in female mice. Indeed, in female mice, only 36% of kisspeptin neurons in the RP3V send projections to GnRH neurons, suggesting that a significant proportion of the kisspeptin neurons are implicated in other functions (240). Transgenic female mice incapable of GnRH secretion failed to show male-directed preferences. A single subcutaneous injection of GnRH restored their physiological behavior, whereas a subcutaneous injection of kisspeptin-10 failed to elicit a male-directed preference in these mouse models (240). Collectively, these data suggest that olfactory mate preference in female mice is mediated through RP3V kisspeptin via GnRH neurons.

Exposure of ovariectomized goats to a sexually mature male goat increases arcuate kisspeptin neuronal activity which leads to simultaneous LH pulse generation (242). Anestrous ewes introduced to a male potential sexual partner, display increased c-Fos activity within the arcuate kisspeptin neurons with concomitant rise in LH amplitude and pulse frequency. Interestingly, this effect is abolished when the female ewes are pre-administered a kisspeptin antagonist (243).

Beyond olfaction, kisspeptin also integrates auditory pathways regulating reproductive behavior in female mice. For instance, males emit song-like ultrasonic vocalizations (USVs) to exhibit sexual intentions and attract a receptive female. When female mice were exposed to an audio file of repeated male USVs for 20 minutes, there was an increase in arcuate kisspeptin neuronal activity compared to control noises. Interestingly, exposure to male USVs did not lead to an increase in the RP3V neuronal activity (244). Putting these findings together, it appears that kisspeptin neurons signaling plays a crucial role in conveying olfactory cues in both female and male non-primates, to stimulate opposite sex-directed partner preference and increase LH responses, ultimately maximizing reproductive success. The brain regions involved in non-primate olfactory partner preference include the MePd, the RP3V and the ARC. Additionally, in female mice, acoustic male cues appear to be related to the arcuate (rather than RP3V) kisspeptin neurons.

#### Pre-copulatory and copulatory behavior in non-human species

Following sexual mate selection, rodents engage in distinct copulatory actions. In males, these encompass mounting, thrusting, intromission, and ejaculation (245). Female rodents typically control the initiation and timing of copulatory contacts to ensure synchronization with ovulation and thus optimize probability of successful fertilization (246). Once partner preference is established, females display receptive behaviors, such as lordosis, essential for intromission (247).

The MeA, a key area in mediating male copulatory behaviors (e.g., erections and intromission), expresses androgen receptors, yet direct administration of androgen in the MeA did not induce spontaneous erections, thereby implicating involvement of other pathways (248). Notably, bilateral radiofrequency ablation of the MePD in reproductive-age male rats abolished non-contact erection (ex-copula) observed when males are placed in proximity to inaccessible estrous females. However, normal male copulatory behavior remained intact when placed with receptive females, albeit with significantly longer intervals between intromissions (249). Consistently, intracerebral microinjections of kisspeptin directly into the MeA stimulate ex-copula erections in a dose-dependent manner, an effect blocked by pre-treatment with a kisspeptin receptor antagonist (250). These findings suggest that kisspeptin signaling within the MeA mediates ex-copula, while other factors likely contribute to behaviors observed upon opposite-sex sexual contact and copulation. Furthermore, kisspeptin’s effects on ex-copula erections appear to be specific to the MeA, as demonstrated by the absence of any discernible effects when kisspeptin is administered intracerebroventricularly, despite comparable LH responses (250).

In female rodents, the RP3V, which is also central to sexual motivation and partner selection, influences pre-copulatory and intra-copulatory behaviors (240). Ablation of 70% of kisspeptin neurons in the RP3V impaired lordosis, which is rescued by a single peripheral kisspeptin-10 injection (240). Consistently, optogenetic stimulation of RP3V kisspeptin neurons during male mounting enhanced lordosis. Utilizing female mice with genetic manipulation resulting in the absence of GnRH secretion during adulthood demonstrates that while male-directed preference is eliminated in the absence of GnRH signaling, lordosis behavior remains unaffected (240). This suggests that lordosis operates independently of GnRH signaling.

Tracing studies identified a subset of nitric oxide synthase (NOS) expressing neurons in the ventromedial hypothalamus (VMHvl) that form communications with RP3V kisspeptin neurons. Kisspeptin-10 injection directly into the VMHvl induced a significant increase in lordosis, whilst administration into the PVN had no significant effect on lordosis expression (251), thereby confirming site specificity for kisspeptin’s actions. Consistent with the concept that nitric oxide (NO) is a key neurotransmitter downstream of kisspeptin neurons, nNOS knockout female mice (nNOS^−/−^; OVX+E+P) experience attenuated lordosis, not restored with subcutaneous kisspeptin-10 injection. By contrast, *Kiss1*^−/−^ (OVX+E+P) females injected with the NO-donor, SNAP, showed wild type-levels of lordosis. Furthermore, bilateral administration of the nNOS inhibitor, L-NAME, in the VMHvl of female mice led to a strong deficit in lordosis behavior. Consistently, SNAP administration into the VMHvl induced a significant increase in lordosis behavior (251). Taken together, these findings highlight NO as a key neurotransmitter downstream of kisspeptin neurons mediating both mate preference and lordosis behavior (240).

All in all, in female rodents, sexual partner preference seems to depend on downstream GnRH-signaling (240). However, lordosis behavior, while independent of GnRH-signaling, relies on the synergistic action of NO within the ventromedial hypothalamus (251).

### Kisspeptin in human sexual behavior

While there are no direct reports of sexual behaviors in humans with kisspeptin variants, animal studies have provided valuable insights discussed previously in the manuscript. For instance, *Kiss1* knockout male rats exhibited reduced sexual behavior, irrespective of sex-steroid milieu (233), which was restored upon kisspeptin replacement (238). Similarly, *Kiss1* knockout female mice failed to show male-directed preference in mate choice tests, a behavior also rescued by kisspeptin administration (240). These animal studies suggest a significant role for kisspeptin in regulating sexual behavior, and further research in humans would be highly informative

Much like in non-human species, in humans, kisspeptin plays an important role in modulating signaling pathways involved in attraction, desire and arousal through integration of external stimuli, such as olfactory and facial recognition cues, thereby regulating sexual attraction and reproductive behavior towards potential mates (252). The primary olfactory network in human projects to key limbic areas involved in sexual and emotional processing (253) and functional neuroimaging studies indicate that exposure to feminine scents increases brain activity in limbic regions associated with sexual desire and arousal in heterosexual men (254).

A randomized, placebo-controlled study employing functional neuroimaging, hormonal assessments, and psychometric evaluations revealed that intravenous kisspeptin-54 (1 nmol/kg/hr over 75-minutes) in healthy heterosexual men exposed to a validated pleasant feminine scent delivered nasally (Chanel No5) resulted in heightened brain activity within olfactory and limbic circuits, including the amygdala and thalamus, compared to vehicle administration (255). These activated regions are recognized for their involvement in olfactory processing, the hedonic valuation of olfactory stimuli, and sexual arousal in humans. Notably, kisspeptin exhibited no impact on brain activity in control motor areas, thereby highlighting the specificity of its effects within olfactory and limbic circuits associated with sexual behavior in men exposed to feminine olfactory feminine cues (255).

Similarly, during a facial attractiveness task, kisspeptin-54 selectively increased activity in frontal brain regions implicated in human perception of beauty, including the medial pre-frontal cortex (mPFC) and superior frontal gyrus in response to attractive female faces (255). Importantly, kisspeptin-54 activated aesthetic brain regions, such as the anterior cingulate cortex (ACC) and insula, more robustly in men with lower baseline sexual quality of life (255). These regions are implicated in sexual arousal, facial attraction, and motivation towards reward, thereby suggesting that the enhanced effects of kisspeptin in these individuals might serve to accentuate attraction and motivation to engage in sexual contact, particularly in individuals experiencing lower sexual quality of life (255). Crucially, all these effects occurred in the absence of any downstream sex-steroid changes. Therefore, this data emphasizes the targeted region-specific effects of kisspeptin, contingent on the nature of the attraction cue, whether it be olfactory or visual (255).

Kisspeptin also seems to be implicated in enhancing sexual desire and arousal in humans, in response to external erotic stimuli (256). In a functional neuroimaging study, intravenous kisspeptin-54 was administered to healthy heterosexual men while viewing erotic images. This led to enhanced brain activity in the anterior and posterior cingulate as well as the left amygdala during kisspeptin administration compared to placebo (256). These regions are known to express kisspeptin and kisspeptin receptors (14,15), and are areas that are known to be activated by sexual stimuli in humans (257,258). Furthermore, the left amygdala has been well described as the center involved in sexual and emotional processing in men. The activations observed correlated well with responses to psychometric questionnaires, providing further functional relevance of these findings. An inverse correlation between the degree of sexual-processing network enhancement (including the cingulate, putamen, and globus pallidus), and the level of aversion to sex that the men displayed was evident, suggesting a role for kisspeptin in sexual disinhibition. Moreover, in response to erotic stimuli, kisspeptin enhanced activity in limbic structures (including the hippocampus, amygdala, and cingulate) more in men with lower baseline reward behavioral scores. As human sexual behavior is closely associated with pleasure and reward, this implies that kisspeptin’s effects might be accentuated in individuals experiencing psychosexual disorders where reward behavior may be suppressed.

In mammalian reproduction, emotional attachment towards a partner often serves as a prerequisite for engaging in sexual encounters, a phenomenon commonly referred to as ‘bonding’. Bonding can manifest in various forms, including romantic love, maternal love, or unconditional love. The role of kisspeptin in romantic love bonding has been studied in humans using functional magnetic resonance imaging (fMRI) (256). In heterosexual healthy men intravenous kisspeptin-54 modulated the response to couple-bonding images in regions similar to those activated in response to sexual images, such as the anterior and posterior cingulate and the amygdala (256). Kisspeptin-54 also enhanced activity in the thalamus and globus pallidus known to express kisspeptin and kisspeptin receptors in humans and were regions not previously activated in response to sexual images but are implicated in “romantic love” (259). Importantly, the enhancement of amygdala activity, also associated with bonding behaviors (259), in response to the bonding images, correlated with reported improvements in positive mood (256). All in all, these data demonstrate that kisspeptin plays an important role in processing both sexual and bonding stimuli in humans, both of which are essential drivers of sexual behavior. Furthermore, a study using the same administration protocol in healthy heterosexual men showed that kisspeptin did not modulate brain regions in response to visual food stimuli or appetite parameters, as assessed by fMRI and psychometric tests, respectively (260).This is significant as it proves that kisspeptin’s actions on the limbic system are confined to sexual and emotional stimuli. Collectively, these data suggest that kisspeptin not only enhances activation in established structures of sexual arousal and bonding, but that this activation also correlates with behavioral measures of reward, drive, and sexual aversion.

To derive further mechanistic insights on how kisspeptin integrates external cues to modulate sexual behavior in humans, it is important to understand kisspeptin’s effects on resting functional connectivity in humans and how this correlates with subsequent processes occurring in response to sexual and emotional stimuli. Therefore, a further fMRI study employing the same experimental protocol, demonstrated that peripherally administered kisspeptin-54 modulates the default mode network (DMN), known to be associated with social and emotional internal processing, and frequently disrupted in psychosexual dysfunction. Importantly, DMN activations correlated with subsequent enhanced activity in limbic brain regions, including the globus pallidus and cingulate gyrus, in response to visual sexual images. Additionally, in response to kisspeptin, enhancement of the DMNs was stronger in individuals with lower reward drive and resulted in reduced sexual aversion (261).

Expanding upon these discoveries, considering that kisspeptin not only enhances activity in sexual and emotional brain centers but also appears to be more effective in individuals with lower quality of life or sex-drive, the investigation into kisspeptin's impact on patients with psychosexual dysfunction has ensued (discussed in later sections). Recognizing desire for sexual stimulation as a fundamental element of the human sexual response, these findings from rodents to humans pave the way for potential clinical utilization of kisspeptin in addressing such issues.

### Translational utility of kisspeptin in hypoactive sexual desire disorder

Hypoactive sexual desire disorder (HSDD) is a common cause of reduced sexual desire in both men and women, impacting approximately 8% of men (262) and 10% of women (263). In both sexes, HSDD manifests as low sexual desire with associated distress and significant impact on quality of life. Presently, there are no licensed pharmacotherapies available for men or post-menopausal women, while treatments for pre-menopausal women such as bremelanotide and flibanserin are limited by their effectiveness or side effects (264). While meta-analysis of randomized controlled trials has shown small but positive effects of testosterone in postmenopausal women with HSDD, testosterone’s impact on individuals’ wellbeing, musculoskeletal, and cognitive health, as well as its long-term safety in women, warrant further research (265). Thus, there exists a pressing clinical need for novel, safe, and effective therapies to address the considerable burden posed by HSDD.

HSDD is characterized by the top-down theory, which posits that increased activity in higher cortical, cognitive brain regions typically involved in introspection, self-criticism and guilt inhibits lower limbic and emotional regions associated with sexual desire and arousal (266). In a study involving heterosexual men with HSDD, intravenous kisspeptin-54 administration modulated brain regions known to express the kisspeptin receptor in humans when exposed to erotic videos. Specifically, kisspeptin enhanced activity in sexual arousal centers, including the left ACC, as well as the left middle frontal gyrus (MFG), a recognized executive attention center. Furthermore, kisspeptin administration led to a significant deactivation of brain regions involved in self-monitoring and introspection, such as the bilateral parahippocampus, frontal pole and precuneus (267). Remarkably, kisspeptin administration led to a 56% increase in penile tumescence (akin to sildenafil), compared to placebo, while watching erotic videos. Additionally, male participants reported increased ‘happiness about sex’ in response to kisspeptin. Notably, all the aforementioned effects of kisspeptin occurred despite stable testosterone levels, which typically rise much later in response to kisspeptin (267). Hence, kisspeptin's capacity to heighten sexual arousal and penile tumescence in heterosexual men with HSDD may stem from its capability to deactivate brain regions responsible for self-monitoring and self-control. This deactivation, in turn, alleviates inhibitory constraints on sexual arousal centers in the brain, thereby intensifying arousal levels.

Similarly, kisspeptin demonstrated efficacy in pre-menopausal women with HSDD (264). When viewing erotic content, intravenous kisspeptin-54 infusion in women with HSDD resulted in the deactivation of higher cortical brain centers, such as the left inferior and middle frontal gyri. These areas are implicated in the ‘internal monologue’, feelings of guilt and inhibitory control and are hyperactivated in women with HSDD. Deactivation of the left inferior frontal and middle frontal gyri by kisspeptin can therefore allow lower-level responses to be expressed. Indeed, women with HSDD also experienced hyperactivation of brain centers involved in sexual arousal following kisspeptin administration, including the supramarginal and postcentral gyri. Kisspeptin-induced enhancement of the posterior cingulate activity correlated with reduced sexual aversion to male faces. Women experiencing higher distress levels regarding sexual function at baseline showed more enhanced brain activity in sexual centers, including the hippocampus, when exposed to erotic stimuli. Kisspeptin administration also deactivated the temporoparietal junction associated with negative perception of others and reduced self-consciousness in response to viewing male faces (264).

In both men and pre-menopausal women with HSDD, kisspeptin appeared to modulate brain regions known to express the kisspeptin receptor, suggesting potential direct receptor-mediated actions of kisspeptin in these brain regions in humans. Interestingly, kisspeptin also demonstrated effects in brain regions lacking identified kisspeptin receptors in humans, indicating additional indirect mechanisms. Taken together, this suggests that kisspeptin can act directly or indirectly on various brain regions to essentially decrease activity in higher brain centers to relieve the inhibition on downstream sexual brain areas, ultimately enhancing sexual behavior. Importantly, all the aforementioned behavioral kisspeptin trials employing fMRI techniques and assessing sexual behavior in men and pre-menopausal women have utilized kisspeptin-54, as opposed to kisspeptin-10. Kisspeptin-54’s ability to cross the blood-brain barrier (268), and thus having access to act directly on deep brain structures expressing *KISS1R* could be highly relevant for this action (256).

### Mechanistic insights and future directions

Current evidence suggests that kisspeptin might act directly via kisspeptin receptors or interact with various neurotransmitters and signaling pathways to modulate reproductive behavior, mood, and cognition across different species. These include dopamine (236), serotonin (269), glutamate (270), nitric oxide (240) and gamma-aminobutyric acid (GABA) (271). Notably, pre-clinical studies confirm GABAb1 subunit co-expression in the majority of Kiss1 neurons within the MeA and GABAb1 knockout mice have intact *Kiss1* expression in the RP3V and arcuate nucleus, but increased expression in the MeA, the bed nucleus of the stria terminalis (BNST) and lateral septum in both adult male and female mice, despite normal sex-steroid levels (237). The MeA and BNST, known for their involvement in anxiety-like behaviors and social interactions, are influenced by GABAergic neurons. Given GABA's role in mood and behavioral disorders, it is possible that GABA mediates some of kisspeptin's effects, possibly through inhibiting *Kiss1* expression in the BNST and MeA (272).

Corroborating data in rodent models, a study in humans using magnetic resonance spectroscopy (MRS), demonstrated up to 16% decrease in total endogenous GABA levels in the ACC (which enhances in response to sexual and couple bonding stimuli (256)) following an intravenous infusion of kisspeptin-54 (1 nmol/kg/hr) in healthy men in the absence of any changes in testosterone levels (271). The magnitude in GABA reduction is similar to that reported in psychological studies with functional significance (in attention-deficit/hyperactivity disorder (ADHD) (272) and in response to pain (273)). Collectively, the aforementioned data suggests the kisspeptin’s effects on stimulation of the ACC and other limbic brain structures in response to sexual and couple-bonding stimuli in humans are, at least in part, mediated through central GABA inhibition. Whether the interaction with GABA is indirect via modulations of other pathways (serotonin, dopamine, vasopressin, glutamate, nitric oxide) or via direct binding to kisspeptin receptors in the ACC is still to be elucidated.

## 4 Pregnancy

### The roles of kisspeptin in the physiology of healthy human pregnancy

The process of embryo implantation and placentation are tightly coordinated both temporally and spatially; indeed, dysregulation of trophoblast invasion underlies the pathogenesis of multiple pregnancy-related disorders. Several factors secreted by the feto-placental unit contribute to the autocrine and paracrine regulation of trophoblast invasion. Kisspeptin, originally identified as a metastasis suppressor in various types of cancer called ‘metastin’, has emerged as a key player in implantation and placentation.

During pregnancy, the placenta is the predominant source of circulating kisspeptin. Once the blastocyst penetrates the endometrium, the trophoblastic stem cells form into an inner layer of undifferentiated cytotrophoblasts, and an outer layer of terminally differentiated syncytiotrophoblasts by fusion of non-migratory villous cytotrophoblasts (274). The extravillous cytotrophoblasts eventually differentiate into the placental bed, responsible for the migration of the embryo into the decidua through degradation of extracellular matrix proteins, and the endovascular trophoblasts, responsible for the uterine spiral artery remodeling (274). *KISS1* is expressed in syncytiotrophoblasts, but *KISS1R* is expressed by both cytotrophoblasts and syncytiotrophoblasts (274). *KISS1R* is expressed at higher levels in first trimester trophoblasts than in trophoblasts from the placenta at term, inferring the importance of kisspeptin signaling in implantation and placentation (275).

Interactions between kisspeptin and cell adhesion molecules promote embryo attachment to the endometrium and upregulate leukemia-inhibitory factor (LIF) to promote stromal decidualization (274). Kisspeptin inhibits placental trophoblast cell migration to regulate implantation and placentation; preventing excessive trophoblastic invasion of the endometrium (275–277). Mechanistically kisspeptin directly activates the ERK1/2 signaling pathway to increase cell adhesion and inhibit first trimester trophoblast cell migration as well as through downregulation of matrix metalloproteinase (MMP) system which inhibits extracellular matrix breakdown (276,277). Kisspeptin-10 stimulated intracellular Ca^2+^ release in primary trophoblasts of the early placenta and subsequently inhibited trophoblast migration, suggesting that this form is the physiological activator of kisspeptin receptor in the human placenta (275). Several studies suggest that kisspeptin may restrain trophoblastic growth by acting as a placental pro-apoptotic agent in a dose-dependent manner (278–280). Kisspeptin is also implicated in uterine spiral artery remodeling and regulating angiogenesis required for placentation, with VEGF-A expression from human placental trophoblasts being downregulated by kisspeptin (274,276,281–283).

**Figure 7:**
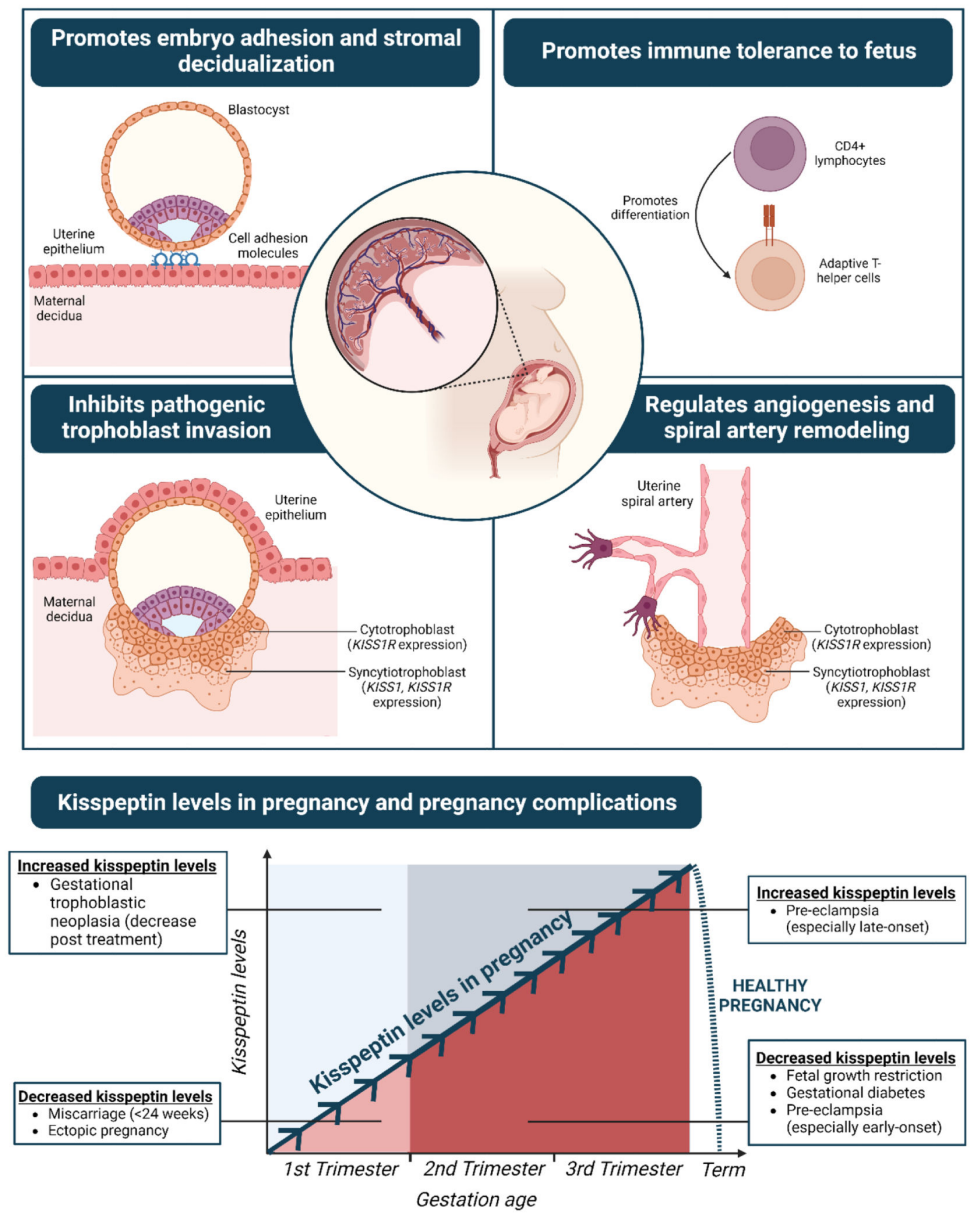
The role of kisspeptin in embryo implantation and prediction of pregnancy complications. Kisspeptin initially promotes embryo attachment to the endometrial epithelium and stromal decidualization through interaction with cell adhesion molecules and leukemia-inhibitory factor (LIF) upregulation. Once the blastocyst penetrates the endometrium, the trophoblast cells form the inner undifferentiated cytotrophoblast cells, and the outer terminally differentiated syncytiotrophoblast cells. Whilst cytotrophoblasts express *KISS1R*, syncytiotrophoblast cells express both *KISS1R* and *KISS1*. Kisspeptin subsequently regulates implantation by preventing excessive trophoblast invasion into the endometrium. Kisspeptin also has roles in angiogenesis, uterine spiral artery remodelling and immune regulation to avoid maternal fetal rejection.

Furthermore, kisspeptin appears to be important in regulating maternal immune tolerance and thus preventing rejection of the developing fetus. *In vitro* studies have demonstrated that there is increased differentiation of naïve human CD4+ lymphocytes into adaptive T-regulatory cells and when incubated with kisspeptin-54 at levels similar to those during pregnancy, with inhibition of T-helper 17 lymphocytes induction (284,285). Natural killer cells also specialize into a regulatory subtype with reduced cytotoxic activity against fetal cells when incubated with kisspeptin-54 (286). Kisspeptin-54 concentrations in pregnancy also reduce the functional activity of neutrophils and increase the functional activity of monocytes; potentially to also facilitate the development of immune tolerance to the fetus whilst balancing the need for maternal immunity (287) (Figure 7).

Kisspeptin has emerged as a promising biomarker to predict several adverse pregnancy complications. Circulating kisspeptin levels increase linearly in healthy pregnancy but are reduced in miscarriage during early pregnancy. Kisspeptin levels are reduced in ectopic pregnancy, fetal growth restriction, gestational diabetes and early onset pre-eclampsia. Kisspeptin levels are raised in pre-eclampsia during the later stages of pregnancy and gestational trophoblastic neoplasia. Figure created with BioRender.com.

Plasma kisspeptin levels increase linearly and dramatically during a healthy pregnancy with gestational age, being over 200-fold greater in the third trimester of pregnancy than in non-pregnant women and returning to non-pregnant levels soon after birth (288–290). Circulating kisspeptin levels in healthy pregnancy are influenced by several variables; advanced maternal and gestational age increase circulating kisspeptin levels, whilst Afro-Caribbean ethnicity, smoking, and high BMI were all associated with lower circulating kisspeptin levels during pregnancy (291).

### The roles and potential diagnostic utilities of kisspeptin in disorders of pregnancy

#### Disorders of placentation

##### Pre-eclampsia and hypertensive disorders of pregnancy

Gestational or pregnancy-induced hypertension is defined as hypertension (systolic blood pressure ≥140 mmHg and/or diastolic blood pressure ≥90 mmHg) that occurs after 20-week gestation (292). Pre-eclampsia occurs when gestational hypertension is accompanied by proteinuria, maternal end-organ or utero-placental dysfunction (292). Early onset pre-eclampsia is attributed to poor placentation and impaired uterine spiral arteries remodeling leading to disruption in placental perfusion, whilst late onset pre-eclampsia occurs when the placenta outgrows the uterine circulatory capacity leading to placental malperfusion (293). Both etiologies result in placental hypoxic stress, however fetal growth restriction (FGR) is more associated with early onset pre-eclampsia given the longer duration of dysfunctional uteroplacental perfusion (293).

*KISS1* expression was increased in the placenta of pre-eclamptic pregnancies according to several reports (294–297). Placental *KISS1* expression inhibits trophoblast invasion and angiogenesis with resulting defective remodeling of the uterine spiral arteries, inferring a role in the pathophysiology of pre-eclampsia (298). Conversely, there have also been reports of reduced kisspeptin expression in pre-eclamptic placentae (299). However, *KISS1R* expression has also been found to be increased in pre-eclampsia compared to normal healthy pregnancy and this may facilitate increased functional activity of kisspeptin in pre-eclampsia (299).

Varying levels of circulating kisspeptin have been reported in hypertensive disorders of pregnancy according to the subtype, severity, and timing of onset. Most studies report reduced kisspeptin levels in gestational hypertension compared to normotensive pregnancy with levels declining further with severe disease and in pre-eclampsia (294,300–306), which may reflect reduced placental mass (303,304). Correspondingly, both circulating kisspeptin levels and placental mass are lower in early onset pre-eclampsia compared to late onset pre-eclampsia (303,307,308).

FGR describes both intra-uterine growth restriction (fetal weight <10^th^ centile for gestational age with abnormal umbilical artery doppler flow) and small for gestational age (delivery weight <10^th^ centile for gestational age) (309,310). FGR is postulated to be the result of abnormal trophoblast invasion and spiral artery remodeling leading to deprivation of adequate oxygen supply to the placenta (311,312). Ischemic injury from this inadequate oxygen supply leads to generation of reactive oxygen species, subsequent apoptosis, and restriction of placental and fetal growth (311,312). As kisspeptin has been implicated in regulating trophoblast invasion and spiral artery remodeling, kisspeptin levels have been demonstrated to be lower in FGR across all three trimesters compared to healthy pregnancy (300,308,313,314) and may contribute to the pathophysiology of FGR. The low circulating kisspeptin levels in FGR may be representative of the low placental mass.

However, recently kisspeptin levels have been demonstrated to bear no association to the severity of pre-eclampsia and are increased in hypertensive disorders of pregnancy during the third trimester (308). Likewise, another study found that plasma kisspeptin was higher in late-onset pre-eclampsia (>34 weeks gestational age) than in gestation-matched controls, but this was not observed for early-onset pre-eclampsia (≤34 weeks gestational age) (315). Discrepancies between the findings of existing studies may be attributed to confounding variables such as BMI and gestational age as well as the complexities of different disorder subsets as well as analytical issues (308). These would need to be considered in future larger observational studies to address these inconsistencies, assessing each subset of hypertensive disorders of pregnancy and pre-eclampsia separately throughout pregnancy and using robust assays.

Similarly, the placenta has also been shown to express high levels of *TAC3* (316) particularly in the outer syncytiotrophoblast layer (317), facilitating secretion of NKB into the maternal circulation (318). NKB is also present in cord blood (318), consistent with a potential role in the physiology of feto-placental circulation. High dose NKB infusion in female rats increased uterine weight by 37%; moreover, NKB levels correlated with nitric oxide metabolites, with higher circulating levels observed in women with pre-eclampsia and IUGR (319). Therefore, suggesting that NKB, together with locally secreted metabolites such as nitric oxide, could be important in the hemodynamic adaptation during pregnancy to ensure adequate fetal blood supply. As pre-eclampsia is a disorder of trophoblastic invasion, NKB is postulated to be a potential biomarker of pre-eclampsia, with plasma NKB concentrations becoming detectable by the ninth week of gestation (320). NKB plasma concentrations are low and increase throughout normotensive pregnancies, with highest concentrations at term before reducing postpartum, consistent with NKB being reflective of placental mass (319,321). In contrast, pre-eclamptic women demonstrate a significantly higher level of plasma NKB and NKB placental expression during the third trimester (319,322–325). NKB, as a marker, appears unique to pre-eclampsia and has no demonstrated association with other hypertensive disorders (326). However, whether plasma NKB levels have predictive value in early pregnancy for subsequent pre-eclampsia development needs to be clarified with further longitudinal studies.

##### Gestational trophoblastic disease

Gestational trophoblastic disease (GTD) is characterized by abnormal proliferation of placental tissue, including premalignant complete or partial hydatid moles and malignant gestational trophoblastic neoplasia (GTN). GTN is the result of malignant lesions (327) that arise from placental villous and extravillous trophoblasts, comprising choriocarcinoma, invasive mole, placental site trophoblastic tumor and epithelioid trophoblastic tumor (328). Serum β-hCG levels are utilized for the diagnosis, staging and prognostication of GTN (328,329). *KISS1* and *KISS1R* expression are reduced in choriocarcinoma cells compared to healthy placentae and benign hydatidiform molar pregnancies (330). Reduced KISS1/KISS1R signaling locally may mechanistically result in dysregulation of the placentation process thus facilitating trophoblastic invasion in GTN, and so could have diagnostic utility in distinguishing metastatic from non-metastatic forms of GTD (289). Conversely, circulating kisspeptin levels are elevated in GTN compared to healthy pregnancy likely as a reflection of the increased malignant trophoblastic mass in GTN (289), with levels significantly declining post-chemotherapy.

##### Miscarriage

Miscarriage is the spontaneous loss of an intrauterine pregnancy before 24 weeks of completed gestation (331). Establishing the diagnosis of miscarriage can be challenging, and pregnancy can be failing for some time before pregnancy loss is conclusively confirmed. This prolonged phase of uncertainty and the need for serial investigations poses significant psychological burden (331,332).

Plasma kisspeptin levels, corrected for gestational age, are reduced by 60-79% in pregnancies that ended with miscarriage compared to healthy pregnancies (291,333–337). Likewise, trophoblastic kisspeptin expression was lower in pregnancies that ended with miscarriage compared to those seen following elective termination of pregnancy (338). Plasma kisspeptin levels can reflect the type of miscarriage, with lower levels reported in complete miscarriage compared to incomplete or missed miscarriage (291).

The diagnostic performance of kisspeptin in identifying cases of miscarriage remains high into the late first trimester (>8 weeks gestation), unlike the alternative biomarker β-human chorionic gonadotropin (β-hCG) (291). Previous studies in women undergoing assisted reproduction showed that β-hCG levels were a better predictor of pregnancy outcome than kisspeptin levels in early first trimester (339,340). These findings may be attributed to the very early gestation at which kisspeptin levels were being measured (12-21 days after embryo transfer); indeed, the performance of kisspeptin for predicting miscarriage improves with gestation (291,339,340). A combination approach, utilizing both plasma kisspeptin and β-hCG levels can provide a higher diagnostic accuracy of miscarriage at all gestations (AUCROC 0.92, 95% CI 0.89-0.95) (291,334,336).

##### Ectopic pregnancy

Ectopic pregnancy refers to an embryo implanting and developing outside of the uterine cavity most commonly in the fallopian tubes and therefore poses the risk of potentially life-threatening complication of tubal rupture (341). Ectopic pregnancy is currently diagnosed using ultrasonographic evaluation and serialized serum β-hCG level measurements (342,343). However, about 1 in 5 extra-uterine pregnancies have a β-hCG rise in keeping with an intra-uterine pregnancy. Furthermore, a viable intra-uterine pregnancy may not be visualized on ultrasound if β-hCG levels are less than 1000 IU/L (342). Kisspeptin has been investigated as a potential biomarker of ectopic pregnancy. Kisspeptin levels are lower in ectopic pregnancy than healthy pregnancy but are higher than the levels seen in miscarriage (344). Lower circulating kisspeptin levels at early gestational ages can challenge detection and therefore, larger studies are needed to determine the utility of kisspeptin for diagnosing ectopic pregnancy at early gestational ages.

##### Gestational diabetes mellitus

Gestational diabetes mellitus (GDM) affects 20% of pregnancies worldwide (345). During pregnancy there is a physiological increase in maternal insulin resistance to provide glucose to the developing fetus; GDM occurs when maternal pancreatic β-cells fail to adapt to this demand (346,347).

*In vitro* and *in vivo* studies have revealed various physiological effects of kisspeptin in glucose-dependent pancreatic β-cell regulation. Kisspeptin-10, kisspeptin-13 and kisspeptin-54 all potentiate glucose-stimulated insulin secretion (GSIS) in mouse and human islet cells *in vitro* (348–351). At lower glucose concentrations (2.8-11.1 mmol/L) kisspeptin-13 and kisspeptin-54 paradoxically demonstrate a dose-dependent inhibition of insulin secretion in mouse islets, an effect not seen at higher glucose concentrations (352). Kisspeptin-54 has also been shown to increase GSIS *in vivo* when administered to healthy men following an intravenous glucose tolerance test (353). In addition, chronic administration of kisspeptin-10 to non-pregnant mice potentiated GSIS and improved glucose tolerance (354). These data suggest that kisspeptin can enhance GSIS and glucose tolerance in the context of hyperglycemia.

Indeed *Kiss1r*-null female mice demonstrated impaired glucose tolerance, hyperlipidemia, and weight gain (355). β-cell specific *Kiss1r* knockout and pharmacological inhibition of *Kiss1r* causes reduced β-cell proliferation compared to what is expected in murine pregnancy, thus reducing GSIS and impairing glucose tolerance (354). Nonetheless β-cell mass is not reduced to pre-pregnancy levels, suggesting that kisspeptin mediated pathways are not the sole signal to drive β-cell proliferation during pregnancy (354).

In GDM, human placental *KISS1* and *KISS1R* expression is elevated in the third trimester (356,357), however, circulating kisspeptin levels are either reduced (301,354) or not significantly altered (308,358). Significant positive correlations between kisspeptin and AUC plasma insulin levels following an oral glucose tolerance test, glucose-stimulated insulin levels, and β-cell secretory function have been demonstrated in the third trimester. However, no significant correlations were observed between plasma kisspeptin and fasting plasma insulin or markers of insulin resistance (354). This suggests that the described positive correlation between kisspeptin and glucose-stimulated insulin levels are not attributable to higher circulating glucose levels or altered insulin resistance (354). In this study, women with GDM also had significantly lower plasma kisspeptin levels than women without GDM (354) thus implicating a role for kisspeptin in the adaptive response to glucose homeostasis during pregnancy.

##### Preterm birth

Preterm birth is defined as births before 37 completed weeks of gestation (359). Kisspeptin may play a role in preterm birth and has been proposed to initiate labor through increased oxytocin neuronal firing rates in pregnant rats (360). Circulating kisspeptin levels, adjusted for gestation, are higher in pregnancies affected by preterm birth than in controls during the late-first trimester (308). The adjusted odds of preterm birth are increased by 20% (95% CI, 1-42%) for every 1 nmol/L increase in plasma kisspeptin (308). Placental *KISS1* mRNA expression is increased in preterm placentae than in term placentae, placentae from with term vaginal delivery having a higher *KISS1* mRNA expression than term cesarean delivery (361). This implicates increased placental kisspeptin expression in the induction of labor. However, no differences have been found in the third trimester in circulating kisspeptin levels between pregnancies reaching term and preterm birth (308,361). Further studies are needed to evaluate if there are any differences in kisspeptin levels preceding onset of labor.

## 5 Menopause

### The physiological roles of kisspeptin and NKB in menopause

Menopause marks the permanent cessation of menstruation and reproductive capacity in women and is characterized by depletion of ovarian follicles, reductions in sex-steroids and inhibin B levels. The loss of inhibitory actions of sex-steroids and inhibin result in marked increase in GnRH and downstream gonadotropin activity. As described in previous sections, KNDy neurons are key intermediary neurons which mediate sex-steroid feedback on GnRH neurons; thus, the hypoestrogenic state in menopause leads to increased KNDy neuronal activity.

Evidence from animal studies, largely using ovariectomized models, has furthered our understanding of the neuroendocrine changes in menopause. Ovariectomized cynomolgus monkeys demonstrated marked neuronal hypertrophy and increased NKB and kisspeptin gene expression in the arcuate nucleus (47). Similarly, increased KNDy neuronal cell size was evident in ovariectomized mice (362). Estrogen replacement in these models was able to revert the heightened NKB and kisspeptin gene expression and KNDy neuronal size to their baseline levels (47,362). This indicated that the hypertrophic morphological changes and alterations in KNDy gene expression specifically resulted from estrogen withdrawal as opposed to being part of the ageing process (47).

Morphological changes observed within human hypothalamus post-menopause were first described in 1966 (363). Pioneering studies using postmortem hypothalamic tissues from postmenopausal women demonstrated that *ERα* (364), *NKB, substance P* (365), and *KISS1* mRNA (47) expressing neurons within the infundibular nucleus were hypertrophic.

Importantly *KISS1* and *TAC3* mRNA expression was increased, conversely *prodynorphin* mRNA expression was reduced (48). This expression profile suggests increased stimulatory action of kisspeptin and NKB and reduced basal inhibitory action from dynorphin thus resulting in increased KNDy neuronal activity and downstream GnRH and gonadotropin secretion following menopause (48).

### The physiological roles of kisspeptin and NKB in menopausal hot flashes

Vasomotor symptoms (VMS), a collective term to describe hot flashes and sweats, are commonly experienced by over 75% of women during the menopausal transition (366). The average duration of symptoms is 7 years (367), but symptoms last longer in a third of women, with 10% of women experiencing symptoms for up to 12 years (368).

In rodent models, the tail functions as the primary heat exchange organ, thus measurement of skin-tail temperature (369) was used as a model to portray cutaneous vasodilation, the primary mechanism of hot flashes in humans (370). In ovariectomized rodents with low E2 levels, the skin-tail temperature is increased to facilitate heat dissipation (371) and the temperature threshold at which heat-defence mechanisms are activated is reduced (372); both mechanisms to facilitate heat exchange.

**Figure 8:**
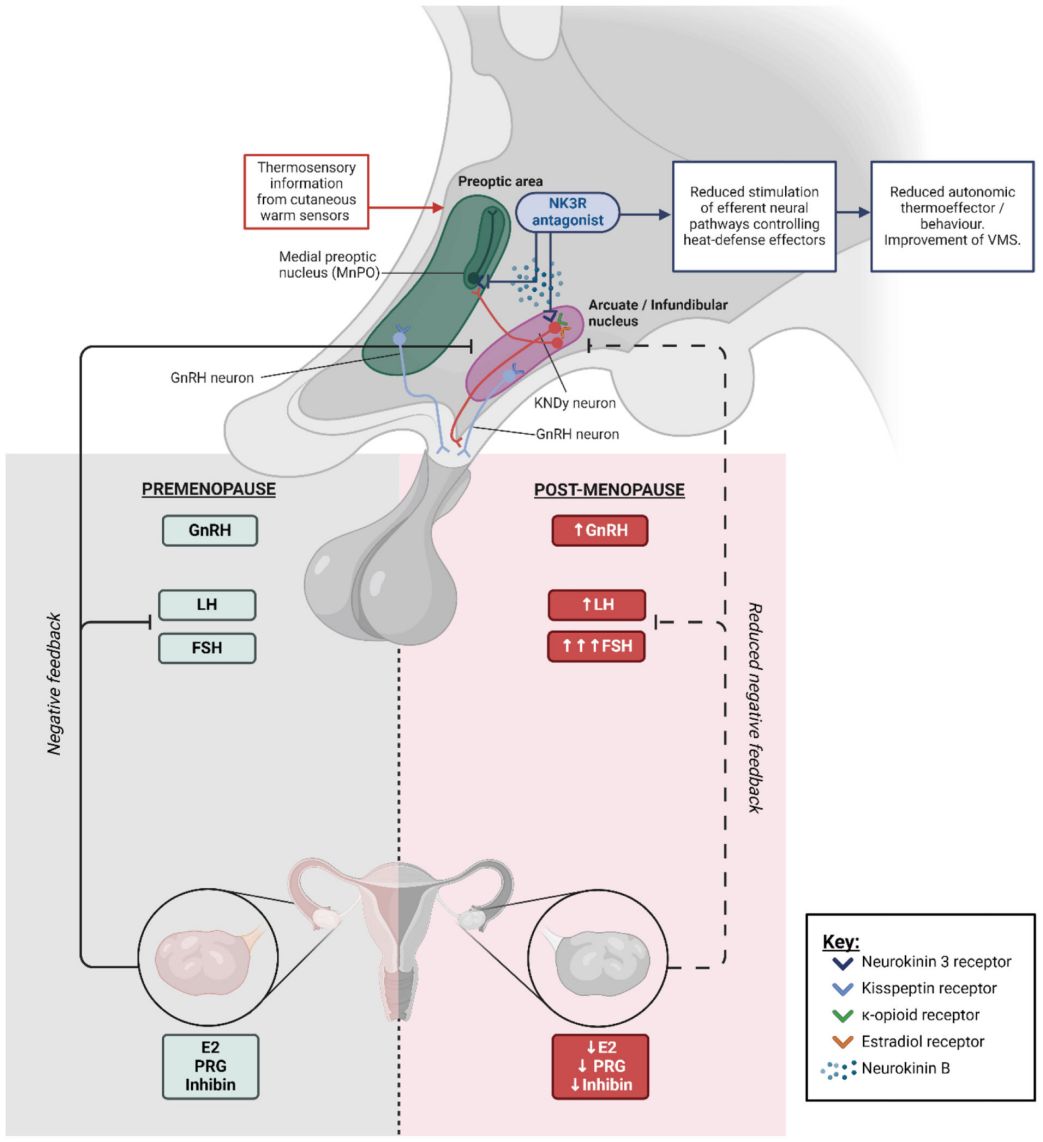
Neuroendocrine changes in menopause and the pathogenesis of vasomotor symptoms. Prior to menopause, there is negative feedback of gonadal sex-steroids on gonadotropins and upstream GnRH. Following menopause, cessation of ovarian function and depletion of ovarian follicular activity leads to a reduction in gonadal sex-steroids and inhibin B production from the ovaries with a corresponding increase in hypothalamic GnRH and gonadotropins from the anterior pituitary gland due to the loss of negative feedback.

Ablation of KNDy neurons led to consistently decreased tail skin vasodilatation supporting the role of KNDy neurons in modulation of body temperature in rodents (369). Anatomically, in preclinical models, arcuate KNDy neurons project to key preoptic thermoregulatory areas including the median preoptic nucleus (MnPO) and medial preoptic area (MPA) (370). The MnPO and MPA integrate thermosensory information from warm-sensitive cutaneous sensors and ultimately influence thermoeffectors such as autonomic cutaneous vasodilation and cold-seeking behavior (370). Furthermore, NK3R expression has been demonstrated in the MnPO. Taken together this intricate anatomical and functional connection between the arcuate nucleus and preoptic area suggests that a possible mechanism underlying the pathogenesis of hot flashes in menopause occur at the level of the hypothalamus through increased NKB signaling (370) (Figure 8).

Within the arcuate nucleus (analogous to the infundibular nucleus in human), KNDy neurons have hypertrophic morphology compared to in the premenopausal state. Due to the loss of negative feedback, there are compensatory increments in the secretion of kisspeptin and NKB and reduced basal inhibitory action from dynorphin. In preclinical models, KNDy neurons project to the hypothalamic median preoptic nucleus (MnPO), which is important for integration of thermosensory information from warm-sensitive cutaneous sensors, mediation of efferent neural pathways controlling heat-defense effectors and ultimately control of thermoeffectors such as autonomic cutaneous vasodilation and cold-seeking behavior. NK3R are expressed by neurons within the MnPO and arcuate nucleus. Thus, antagonism of these NK3Rs offers a novel therapeutic option for the management of VMS.

E2, estradiol; GnRH, gonadotropin-releasing hormone; FSH, follicle stimulating hormone; KNDy; kisspeptin, neurokinin, dynorphin; LH, luteinizing hormone; MnPO, median preoptic nucleus; NK3R, neurokinin 3 receptor; PRG, progesterone; VMS, vasomotor symptoms. Figure created with BioRender.com.

Focal microinfusion of a selective NK3R agonist, senktide, into the hypothalamic MnPO resulted in a rapid, dose-dependent reduction in core temperature (373). Additionally, administration of NK3R agonist increased skin-tail temperature and reduced core temperature in mice, in keeping with heat dissipation effector activation (374) these effects are particularly pronounced in hypoestrogenic states e.g. post-ovariectomy (375). Furthermore, administration of a neurokinin receptor antagonist cocktail (with affinity for NK1R, NK2R and NK3R) into the MnPO prevented the increase in skin-tail temperature heat dissipation response in mice (375). These findings highlight that the NKB/NK3R/NK1R signaling pathway plays a key role in the pathogenesis of hot flashes, and therefore antagonizing the action of NKB in these signaling pathways is delivering a novel therapeutic avenue for VMS (376).

Some studies have shown that there is a close temporal relationship between the onset of VMS and LH pulsatile secretions (377), and significant positive correlations between skin temperature measurements and circulating LH levels (378), however data to support this notion is sparse with small number of subjects included. Women with previous hypophysectomy and FHA with low circulating concentrations of serum gonadotropins challenged this dogma and provided clinical evidence for the uncoupling of LH pulses from the onset of VMS (379). Women with isolated gonadotropin deficiency (representative of defective GnRH secretion or function), experienced similar rates of hot flashes compared to postmenopausal women thus suggesting that alteration of GnRH synthesis and/or release is not involved in the generation of hot flashes (380). Indeed, blinded deconvolution and Bayesian spectrum analysis demonstrated no clear association between LH pulse and hot flashes interval and challenged the long-held dogma regarding the synchronicity of hot flashes and LH pulses (381). Finally, women affected by FHA (representative of dysfunctional neurotransmitter input to GnRH neurons), despite marked hypoestrogenism, reported no symptoms resembling hot flashes (380). These findings substantiate the hypothesis that menopausal VMS occurs secondary to estrogen withdrawal and is mediated through functional changes of estrogen-sensitive afferent intermediary neurons such as KNDy neurons that provide input and regulate GnRH neuronal secretion.

To evaluate the contribution of NKB/NK3R pathway in reproduction, peripheral intravenous NKB infusion was administered to healthy men and premenopausal women during the follicular phase (382). In healthy men and women, NKB infusions did not significantly alter downstream serum gonadotropin or sex-steroid levels compared to vehicle (383). In addition, co-administration of kisspeptin-54 and NKB resulted in significantly lower increases in gonadotropins compared with kisspeptin alone or when kisspeptin-54 and naltrexone were co-administered (384). Intriguingly at higher doses, three subjects experienced a hot sensation and appeared flushed with mild increased in heart rate which stopped promptly following cessation of NKB infusion. Indeed, this was the first observation providing direct evidence of NKB stimulation and induction of hot flashes in humans and ignited interests in antagonizing this pathway as a potential treatment for menopausal hot flashes.

Following the above study, the ability of NKB to elicit hot flash-like episodes was tested in premenopausal women. Intravenous NKB induced hot flashes, both subjectively and objectively (mean heart rate, skin temperature and skin conductance increased by similar magnitudes to changes observed during natural menopausal flashes) (382). Meta-analysis of three genome-wide association studies (GWAS) of postmenopausal women of European American, African American, and Hispanic American descent identified 14 single-nucleotide polymorphisms (SNPs) associated with VMS (385). Intriguingly, these SNPs were all located on chromosome 4 in the *TACR3* locus, which suggested that genetic variation in *TACR3* may contribute to an increased risk of VMS (385). Collectively, these data provided functional and genetic evidence to support the pivotal role of NKB signaling as a mediator of menopausal flushing and sparked interests in antagonism of the NKB through the use of NK3R antagonists to alleviate VMS (376).

Substance P is the endogenous ligand that activates NK1R (365). Central infusion of substance P in mice led to sleep disturbance, an effect blocked by NK1R antagonist pre-treatment (386). Similarly, intravenous infusion of substance P in healthy men led to worsened mood, increased rapid eye movement (REM) latency, and disturbed sleeping patterns (387). Furthermore, substance P infusion caused hot flashes (388) and NK1R has been demonstrated to facilitate heat dissipation through active vasodilatation (389). Therefore, NK1R antagonism may offer beneficial effects not only on the control of VMS but could also improve sleep quality which is frequently disrupted following menopause.

### Translational use of NK3R antagonists; a novel treatment for menopausal hot flashes

To date, four novel neurokinin receptor antagonists that target the NK3R and NK1R have been evaluated in human clinical trials; Pavinetant (MLE4901), Fezolinetant (ESN364), Elinzanetant (NT-814) and SJX-653.

The first proof-of-concept trial of NK3R antagonism in postmenopausal women affected by hot flashes was published in 2017 (390). MLE4901 (Pavinetant) was administered to 28 healthy post-menopausal women aged 40-62 years who experienced ≥7 hot flushes per day in a phase 2, randomized, double-blinded, placebo-controlled, crossover trial (390). MLE4901 resulted in a significant reduction in the number and frequency of hot flashes with 45% reduction in subjective report of hot flashes, 41% and 58% reduction in the severity and interference of weekly hot flashes respectively compared to placebo (390). The positive symptomatic relief was appreciable from the second day of treatment (391,392) thus highlighting the potential to induce rapid relief for VMS.

The effects of MLE4901 on LH pulse profile appeared to be heterogenous with one report describing no significant changes in the number of LH pulses but MLE4901 interestingly increased LH pulse amplitude, and improved orderliness of LH pulses, compared to placebo (390). Conversely in another report, MLE4901 treatment for 7 days resulted in reduction in basal LH secretion with an overall suppressive effect of LH secretion (391). No change in E2 (390) or FSH (391) levels were observed. Changes in hypothalamic GnRH pulse release were postulated to account for reductions in LH pulses following administration of NK3R antagonists and that this could independently suppress VMS. Due to a rise in transaminase levels up to 6x upper limit of normal associated with MLE4901 treatment its development has subsequently been discontinued although other NK3R antagonists have emerged.

Fezolinetant (ESN364) is a selective and reversible antagonist of NK3R. Twice daily Fezolinetant significantly reduced total VMS scores, frequency of moderate/severe VMS and led to a 93% reduction in VMS frequency from baseline to week 12 *vs* 46% with placebo (393). At 3hrs post Fezolinetant dose (during peak drug levels), plasma LH levels decreased by 49.8% *vs* 16.4% with placebo relative to baseline consistent with the proposed mechanism of action of KNDy neuron inhibition (393). No effect on E2, FSH and sex hormone binding globulin (SHBG) levels were observed (393).

SKYLIGHT 1, 2 and 4 are phase 3 multicenter trials of Fezolinetant treatment that resulted in significant reduction in VMS frequency and severity compared to placebo (394) with beneficial effects maintained for 52-weeks (395). Interestingly, the MOONLIGHT trial, conducted in Asian women across 48 Asian countries demonstrated no significant difference in moderate-severe VMS frequency and severity with Fezolinetant use compared to placebo (396). This may be due to genetic differences and environmental influences.

There were no significant changes from baseline endometrial thickness between Fezolinetant and placebo-treated participants, and no effect on bone health after a year of treatment (397). Notably, hepatic safety profile was assessed in participants with relevant hepatic risk factors such as obesity and non-alcoholic fatty liver disease, and there were no evidence of liver function impairment or liver-associated symptoms, including no Hy's law cases to indicate drug-induced liver injury following Fezolinetant use (397).

Elinzanetant is a dual NK1R and NK3R antagonist. Given its dual NK1R/NK3R antagonism it has the potential to decrease GnRH pulse frequency by blocking the effects of endogenous substance P and NKB on the reproductive axis, as the cognate receptors for substance P and NKB are NK1R and NK3R respectively (226). Although NK1R has been proposed to contribute to the pathogenesis of VMS, the predominant action on VMS associated with Elinzanetant is likely to be mediated via NK3R antagonism as NK1R antagonism alone is unlikely to fully alleviate VMS but may provide additional benefits on sleep and anxiolytic effects in postmenopausal women (398). Higher dose regimens of Elinzanetant were associated with significant reductions in VMS frequency (150mg: 84% reduction, 300mg: 66% reduction, placebo: 37% reduction) and in night-time awakening due to night sweats (150mg: 81% reduction, 300mg: 63% reduction, placebo: 32% reduction) at the end of the 14 day period (399) with significant improvements in sleep and quality of life with return to baseline 4 weeks after discontinuation of treatment (400). The OASIS 1 and 2 trials evaluated the efficacy and safety of Elinzanetant 120mg for the treatment of VMS. Elinzanetant use resulted in significant reductions in VMS frequency (55-67% reduction) and severity, evident by 4 weeks of treatment. Furthermore, Elinzanetant improved menopause-related quality of life and reduced sleep disturbances by the end of 12 weeks. (401).

SJX-653 resulted in a reversible suppression (up to 70%) of gonadotropins and testosterone after a single oral dose (dose range 0.5-90mg) in healthy men aged 18-45 years (402). As other NK3R antagonists after achieving similar reductions in LH and testosterone in men have subsequently been shown to demonstrate reduction in menopausal hot flushes, this led to the extrapolation that SJX-653 may be an effective treatment of menopausal hot flushes (402). Subsequently, this prompted a phase 2 trial to assess the efficacy of SJX-653 in postmenopausal women with moderate to severe VMS (403). However, as the primary outcome of safe and efficacious treatment of VMS was not met, the trial was terminated early and its further development since discontinued (403).

Data from animal models and post-mortem hypothalamic studies from postmenopausal subjects paved our understanding of the role of the NKB pathway in the pathogenesis of VMS. Antagonism of the NKB pathway resulted in rapid and sustained relief of VMS and improvements in sleep quality, and overall markers of quality of life. Antagonism of the NKB pathway demonstrates clear effectiveness for the management of menopausal hot flushes and is likely to provide an effective, non-hormonal treatment strategy for the management of VMS. Recently in 2023, the US Food and Drug Administration (FDA) has approved Fezolinetant for use to reduce the frequency and severity of post-menopausal hot flashes marking the first translational application of therapeutic agent targeting the NKB pathway (376).

## 6 Reproductive Bone Health

### Reproductive hormones and bone homeostasis

Skeletal homeostasis in mammals refers to the dynamic equilibrium whereby bone formation and bone resorption are meticulously balanced to maintain stable and optimal bone mass. This equilibrium is essential for promoting growth and ensuring the skeleton’s resilience to mechanical stressors and thereby preventing fractures. Skeletal homeostasis is achieved by the process of bone remodeling which comprises two essential components; osteoclastic bone resorption and osteoblastic bone formation.

Osteoclasts are specialized cells that differentiate from hematopoietic stem cells under the control of receptor activator of nuclear factor kappa beta ligand (RANKL) signaling and are responsible for resorbing old or damaged bone tissue. Enzymatic activity during bone resorption releases fragments of type 1 collagen, including C-terminal telopeptide of type 1 collagen (CTX) and N-terminal telopeptide of type 1 collagen (NTX), into the bloodstream, serving as measurable biochemical markers of bone resorption. On the other hand, osteoblasts differentiate from mesenchymal stem cells, and are the bone forming cells involved in new bone matrix synthesis and mineralization to achieve bone strengthening and facilitate microdamage repair. Osteoblasts release proteins including osteocalcin and cleavage fragments from type 1 procollagen synthesis, such as procollagen type 1 N-terminal propeptide (P1NP), which can be detected in the circulation as biochemical markers of bone formation. Similarly, bone specific iso-enzymes such as alkaline phosphatase (B-ALP) are released by osteoblasts to aid bone mineralization and are also measured as markers of bone formation. Once mineralization is complete, a proportion of mature osteoblasts differentiate into osteocytes, which reside within the mineralized bone. Osteocytes secrete important hormones and chemicals that regulate bone remodeling, including fibroblast growth factor-23 (FGF23; phosphate regulating hormone), RANKL and sclerostin (an antagonist of the osteoblast activator, Wnt). Osteocytes may also act as sensory cells, translating mechanical cues into signals that stimulate bone formation (404).

Bone remodeling is orchestrated by the coordinated action of hormones, such as parathyroid hormone (PTH), vitamin D, growth hormone, insulin-like growth factor-1, sex-steroids and adequate nutrients (405). Crucially, there exists a well-established connection between the HPG axis and bone health. Bone expresses receptors for the action of several reproductive hormones (405), with the predominant action from testosterone and estrogen (406). One of the main secondary causes of skeletal disease such as osteoporosis are reproductive disorders causing sex-steroid deficiency in women and men. Physiologically, androgens influence bone health by directly binding to androgen receptors on bone or indirectly through aromatization to estrogen (406). Estrogen suppresses osteoclast formation and resorption activity, partly through antagonistic effects on the RANKL pathway (407). Furthermore, estrogen induces secretion of semaphoring-3A from osteocytes, a protein that also reduces bone resorption and increases bone formation (408) and exerts anti-apoptotic effects on osteoblasts (409). Therefore, in the absence of adequate levels of sex-steroids, there is significant bone compromise. Indeed, following menopause, a decline in circulating estrogen enhances bone resorption, which is followed by a lesser increase in bone formation due to coupling, resulting in a net loss of bone (410) such that during early menopause there is up to a 100% increase in bone turnover (411). The significance of estrogen deficiency is also highlighted in eumenorrheic (eugonadal) women with anorexia nervosa exhibiting higher BMD than amenorrheic (hypogonadal) women with lower estrogen levels (T-score -1.2 in eumenorrheic *vs* -2.3 in amenorrhoeic women) (412). Whilst optimal sex-steroids are indispensable for bone health, disruptions to the normal process of bone remodeling can occur even in the presence of normal circulating sex-steroid levels. This is evidenced by peri-menopausal women experiencing declines in BMD (413) and women with prolactin-secreting adenomas exhibiting a higher prevalence of fractures, despite a normal sex-steroid milieu (414). Additionally, women with anorexia nervosa suffer bone consequences due to disturbances in leptin, IGF-1, and cortisol, even in the absence of amenorrhea (and thereby intact sex-steroid levels) (415).

### Kisspeptin and bone homeostasis

Emerging evidence suggests that kisspeptin is also a significant player in bone homeostasis. Individuals with loss of function *KISS1R* variants demonstrated delayed bone maturation in the absence of kisspeptin signaling (6). This finding first marked the potential involvement of kisspeptin in bone homeostasis (6). Similarly, CPP from *KISS1R* gain of function variant was associated with accelerated growth and skeletal maturation (23). Admittedly due to the observed significant alterations in sex hormones associated with these conditions (hypogonadotropic hypogonadism or precocious puberty), it is not possible to assert a direct relationship between kisspeptin itself and the observed effects on bone; nonetheless these observations contributed to the interest in the potential role of kisspeptin in bone homeostasis.

The link between kisspeptin and bone homeostasis is increasingly evident, following studies demonstrating significant *Kiss1/KISS1* and *Kiss1r/KISS1R* expression on key cells involved in bone homeostasis, suggesting possible direct effects of kisspeptin on bone.

#### Kisspeptin expression profiles in osteoclasts and osteoblasts

*KISS1R* has been identified as one of the hypoxia-inducible genes in the osteoclast microarray (416). In fact, in humans, *KISS1R* mRNA is expressed throughout the process of osteoclastogenesis *in vitro*, at multiple differentiation stages, ranging from the CD14-monocyte stage to mature osteoclast stage (417).

The osteoanabolic effects of kisspeptin have been demonstrated predominantly in *in vitro* studies in cultured cell lines or osteoprogenitor cells. Using cDNA microarray technology, *KISS1* gene was first identified in osteosarcoma U-2 cell lines in 2003 (418). Further expression studies in osteosarcoma cell lines, commonly used as osteoblastic model, confirmed moderate *KISS1* mRNA and protein expression in U-2 cell lines, weak expression in Saos-2 cell lines, and absent expression in MG-63 cell lines (419). *KISS1* mRNA expression was most notable in immortalized human fetal osteoblastic cells transformed by expression of SV40 large T antigen (hFOB1.19) (419). Interestingly, weaker *KISS1* expression was associated with greater invasive capability in an osteosarcoma cell line *in vitro*, whilst in osteosarcoma elevated *KISS1 in vivo* was associated with relapse or early metastasis (419). Low level heterogeneous expression of *KISS1* was also detected in primary mesenchymal stem cells (MSC) and MSC-derived osteoprogenitor cells (undifferentiated osteoblastic cells) from healthy donors (420). Furthermore, *Kiss1* and *Kiss1r* are expressed in canine osteosarcoma cell lines (COS, POS) (421). Application of kisspeptin to these cell lines increases COS proliferation and expression of bone remodeling factors, such as RANKL and specific serotonin (5HT) receptor (HTR2a) (421).

#### Kisspeptin’s role in bone homeostasis: in vitro data

Expanding upon these expression profiles, *in vitro* data in rodents has recently confirmed kisspeptin’s involvement in osteoblast differentiation. Kisspeptin-10 induced a dose-dependent upregulation of early osteogenic factors mRNA and protein expression such as distal-less homeobox 5 (Dlx5), runt-related transcription factor 2 (Runx2) and ALP, known to be important in osteoblast differentiation in murine fibroblastic mesenchymal stem cell–like osteoblast cell lines (C3H10T1/2) (422). As kisspeptin-10 only exerts its effects in *Kiss1r* expressing cells, this provided evidence that these effects occur via *Kiss1r* expressed on C3H10T/2 cells (422). In rodents, bone morphogenetic proteins (BMPs) that belong to the transforming growth factor-β superfamily, appear to mediate the osteoanabolic effects of kisspeptin-10 through the activation of transcription factors including NFATc4 (in osteoblasts and the embryonic kidney) (422) and Sp1 (in the embryonic kidney) (423). The BMPs implicated in this pathway to date are BMP-2 (422–424) and BMP-7 (423). Therefore, kisspeptin, at least *in vitro*, may act as an autocrine growth factor with pro-proliferative effects on bone (421).

Further pivotal data on the direct effects of kisspeptin on human bone metabolism have emerged more recently. Immortalized human mesenchymal stem cells (hMSC-TERT4) were incubated with kisspeptin-54 for 7 days, to evaluate the effects on osteoblastogenesis (417). This led to an increase in ALP activity, a surrogate for osteoblast activity, by 41%, therefore suggesting enhancement of osteoblastogenesis in this human cell line. Kisspeptin addition to mature osteoblasts did not alter ALP activity, thereby suggesting selective effects on osteoblast precursors (417).

During bone resorption assays, infusion of kisspeptin with osteoclast monocultures revealed potent antiresorptive effects. Microscopic evaluation of the percentage of eroded surface per bone surface demonstrated a dose-dependent effect ranging from 29.6-48.1% reflective of inhibition of osteoclast activity. Kisspeptin exhibited similar effects in osteoblast/osteoclast co-cultures, which represents a more realistic *in vivo* bone remodeling environment, with suppressive effects ranging from 26.2% to 53.4% (417). To translate kisspeptin’s positive effects on bone homeostasis observed *in vitro*, a tentative indirect comparison with established osteoporosis treatments revealed that kisspeptin’s osteoanabolic effects compare favorably with those of teriparatide (425), and its osteoclastic effects with zoledronic acid (426). To date, no studies have explored whether kisspeptin signaling occurs in osteocytes.

#### Kisspeptin’s role in bone homeostasis: in vivo data

The identification of kisspeptin and its receptor expression in bone, coupled with compelling findings that identify kisspeptin’s role in promoting osteoblastogenesis and inhibiting osteoclastic activity *in vitro*, spurred additional research. Further exploration has provided better insights into kisspeptin’s role in the bone *in vivo*, suggesting promising applications for translational research in addressing disorders of skeletal homeostasis, including osteoporosis of various etiologies.

In both male and female (intact or gonadectomized) rodents, chronic administration of 17β-estradiol has been shown to increase trabecular bone mass through ERα. Intriguingly, the action via hypothalamic ERα seems to have the opposite effect on bone (427). Ablation of ERα in the medial basal hypothalamus (MBH) in Esr1Nkx2-1Cre models, results in a robust bone phenotype characterized by increased BMD in trabecular and cortical bones. Stereotaxic-guided ablation of ERα specifically in the arcuate nucleus (ERαKOARC), mirrored the significant increases in BMD observed exhibiting an impressive up to 700% increase in trabecular bone mass and an 80% increase in bone volume/total volume (BV/TV) in female mice. Importantly, these effects manifest independently of alterations in food intake and other circulating hormones known to have osteoanabolic effects, such as leptin, thyroxine, LH, FSH, testosterone and estrogen. Crucially, even after ovariectomy, female mice subjected to ERαKOARC still demonstrate a 50% increase in BMD, suggesting the presence of an intact brain-bone circuit irrespective of the sex-steroid milieu (427). This finding holds significance, suggesting a potential therapeutic avenue for addressing bone mass loss in women due to menopause or other estrogen deficient states. Similarly, striking increases in bone mass were observed in female mice when ERα was stereotactically ablated in arcuate Kiss1-expressing cells (Esr1Kiss1-Cre) using a Kiss1-Cre-GFP knock-in allele. These sexually dimorphic effects appeared to be independent of high E2 levels. A BV/TV increase of approximately 88% at the distal femur, along with similar changes in the L5 vertebra and the cortical bone mass, were also observed. Importantly, ERα ablation in POMC expressing neurons (Esr1POMC-Cre), which share a common lineage with most *Kiss1* neurons, did not lead to skeletal improvements (427). Collectively, these findings suggest that a female-specific brain-to-bone pathway is mediated by a subset of *Kiss1* neurons. Furthermore, transcriptional profiling demonstrated that the observed effects were mediated via BMP signaling and enhanced osteoblast differentiation (427). This aligns with previous *in vitro* findings on the mechanism of action of kisspeptin in the bone.

A recent study utilized parabiosis and bone transplant methods to reveal that a circulating factor, identified as Cellular Communication Network Factor 3 (CCN3), accounts for the high bone mass observed specifically in females, after the deletion of ERα from arcuate Kiss1 neurons in the Esr1NKx2-1 Cre model (428). CCN3’s osteoanabolic effects were demonstrated *in vitro* in mouse and human skeletal stem cells (SSCs) when administered at low concentrations, as well as *in vivo* in gain-of-function and loss-of-function studies. Importantly, CCN3 is co-expressed with kisspeptin in arcuate KNDy neurons, but its expression in arcuate Kiss1 neurons was only significantly increased in both estrogen-depleted lactating females and lactating dams. In lactating dams, removal of pups (forced weaning) led to reduction in CCN3 expression, thus suggesting that CCN3’s bone-promoting property lessens with cessation of lactation (428). Further studies that will help elucidate its interaction with kisspeptin, and how this might be utilized in post-menopausal bone loss and osteoporosis, are warranted. While the exact underlying mechanisms, beyond CCN3, that promote the high mass bone phenotype in Esr1Nkx2-1Cre, Esr1Kiss1-Cre, and ERαKOARC female mice remain undetermined, the findings of the aforementioned studies further implicate the involvement of Kiss1-Kiss1r signaling in skeletal homeostasis, here demonstrated in lactating mice (427). This complex neuro-skeletal circuit may have evolved as an energy conserving mechanism during periods of negative energy balance to regulate metabolic demands of the bone and to preserve reproductive capacity. While these findings imply a potential link, they do not confirm a direct bone effect attributed to peripheral kisspeptin signaling. In addition to offering deeper insights into the physiology of skeletal homeostasis and the pathophysiology of skeletal disorders, these findings suggest the possibility of pharmacologically targeting kisspeptin pathways in humans to improve bone health.

**Figure 9:**
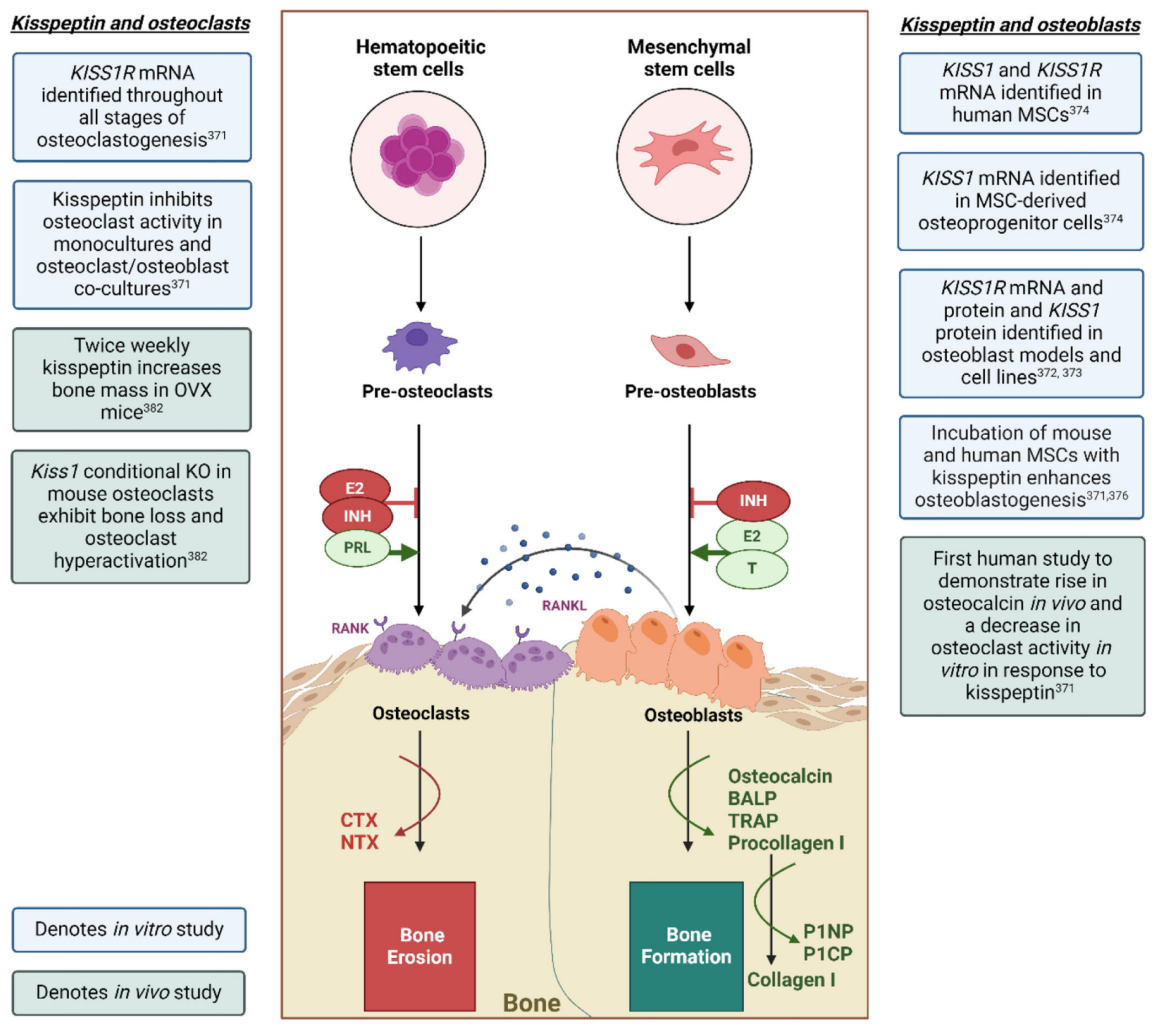
Kisspeptin and bone homeostasis. Schematic diagram illustrating the stages of osteoclastogenesis, from hematopoietic stem cells to mature osteoclasts, and osteoblastogenesis, from mesenchymal stem cells to mature osteoblasts. CTX and NTX serve as biochemical serum markers of bone resorption. Osteocalcin, P1NP, and BALP are produced at various stages of osteoblast action and serve as biochemical serum markers of bone formation. RANKL, secreted by osteoblasts, binds to RANK expressed on osteoclasts, which facilitates bone turnover coupling. This interaction between RANKL and RANK is crucial for osteoclastogenesis and bone remodeling. The blue and green boxes describe the role of kisspeptin in bone homeostasis *in vitro* and *in vivo* respectively. Annotated circles represent different reproductive hormones and their impact on osteoblasts/osteoclasts (inhibitory actions are depicted in red whilst stimulatory actions are depicted in green).

Moreover, a recent study in mice has shown results implicating kisspeptin-10 signaling as a negative osteoclast modulator, thereby conferring bone protective effects. The resorptive effects of osteoclasts rely on the phosphorylation and activation of Src kinase. *In vitro* kisspeptin-10 dose-dependently upregulated the expression of a phosphatase (Dusp18), which phosphorylates and inactivates Src. *In vivo*, both whole-body (*Kiss1*^−*/*−^, *Gpr54*^−*/*−^, *Dusp18*^−*/*−^) and osteoclast conditional knockout (*Kiss1* cKO, *Gpr54* cKO) mice exhibited bone loss and osteoclast hyperactivation (429). These effects were paralleled by increased osteoblast differentiation in the *Kiss1*^−*/*−^ and *Gpr54*^−*/*−^ models. The observed phenomenon may be attributed to a mechanism known as 'bone coupling', wherein the activation of osteoclasts is reliant on cytokines derived from osteoblasts, such as M-CSF and RANKL. Moreover, in ovariectomized mice, both intravenous injections of kisspeptin-10 and bone-targeting kisspeptin-10 ((DSS)*6-Kp-10) administered twice weekly for two months exhibited bone-protective effects (429). The bone-targeting (DSS)*6-Kp-10 was designed using six repetitive sequences of the amino acids aspartate, serine, and serine, ensuring an effective bone surface-targeting delivery system (430). Indeed, the bone-protective effects of (DSS)*6-Kp-10 were far superior compared to those receiving equivalent doses of kisspeptin-10, as determined by Von Kossa staining and parameters of trabecular bone analysis. This difference was observed despite similar gonadotropin levels. Treatment with a higher dose of bone-targeted (DSS)*6-Kp-10 led to increased bone mass in both ovariectomized and gonad-intact mice, as shown using micro-CT analysis, and TRAP staining revealed suppressed osteoclast activation in both groups, supported by measurements of osteoclast parameters. Collectively, these data suggest that the kisspeptin-10/Gpr54 signaling axis plays a bone-protective role and improves bone health both in vitro and in vivo by mechanisms which include inhibiting osteoclastic bone resorption (Figure 9). Kisspeptin-10 could, therefore, be utilized as a potent therapeutic target for the treatment of osteoclast-associated bone loss, such as age-related osteoporosis (429).

BALP, bone alkaline phosphatase; CTX, C-terminal telopeptide of type I collagen; E2, estradiol; FSH, follicle stimulating hormone; INH, inhibin; KO, knock-out; MSC, mesenchymal stem cells; NTX, N-terminal telopeptide of type I collagen; OVX, ovariectomized; P1NP, procollagen type 1 N-terminal propeptide; P1CP; carboxy-terminal propeptide of type 1 procollagen; PRL, prolactin; RANK; receptor activator of nuclear factor kappa-B; RANKL, receptor activator of nuclear factor kappa-B ligand; T, testosterone; TRAP, tartrate-resistant acid phosphatase. Figure created with BioRender.com.

### Potential translational utilities of kisspeptin in bone health

The only study to demonstrate that kisspeptin’s *in vitro* effects on bone metabolism could be translated into humans was performed recently (417). When intravenous kisspeptin-54 was administered to 26 healthy men over 90 minutes, it elicited a 20.3% rise in total osteocalcin and a 24% rise in carboxylated osteocalcin levels, compared to placebo. Carboxylated osteocalcin is the predominant form of osteocalcin involved in bone remodeling. Therefore, this acute rise following only 90 minutes of kisspeptin and in the absence of any sex-hormone changes, suggests potent positive bone effects of kisspeptin *in vivo*. In contrast to the *in vitro* findings where kisspeptin incubation with mature osteoblasts did not increase ALP, the observed osteocalcin rise suggests effects on more mature osteoblasts *in vivo* (417). The short infusion of kisspeptin-54 elicited no discernible impact on bone resorption (CTX) potentially due to the short (90 minute) exposure to kisspeptin. To potentially observe significant alterations in bone turnover markers, it is plausible that sustained administration of kisspeptin-54 or its receptor analogues, TAK448/MVT602, may translate the suppression of osteoclast activity seen *in vitro* and in non-human *in vivo* studies. Further investigations with extended exposure periods and continuous administration may reveal the full spectrum of kisspeptin’s impact on bone turnover. All in all, these findings indicate that acute administration of kisspeptin may have direct beneficial effects on skeletal homeostasis in humans (likely via KISS1R on mature osteoblasts), independent of effects on downstream sex-steroids.

Kisspeptin emerges as a promising therapeutic option for preventing and treating skeletal complications associated with conditions characterized by sex-steroid deficiency, such as hypogonadotropic hypogonadism, POI, and menopause. In women experiencing FHA, BMD is significantly reduced at various skeletal sites, in both trabecular and cortical bone (415). This decline in BMD correlates with an increased risk of stress fractures, extending beyond the effects of estrogen deficiency (415). Notably, reduced kisspeptin signaling is a recognized factor in FHA as described previously. Kisspeptin’s favorable safety profile, along with its potential to restore menstrual cyclicity and potentially ovulation in women with FHA, positions it as a competitive agent for both bone, menstrual health, and fertility in FHA. This advantage is particularly noteworthy as it may aid bone health without the associated side-effects and risks associated with HRT (415). Alternatives to HRT for bone health in these women are limited. Leptin levels are reduced in FHA and although subcutaneous metreleptin over two years was associated with 4-6% BMD gain in the lumbar spine in exercising women, it was also associated with 3% weight loss which has therefore dampened its viability for FHA treatment (431). By contrast, current evidence in humans does not suggest an association between kisspeptin administration and weight loss. Further studies investigating the impact of kisspeptin signaling on the bone in cases of osteoporosis, particularly in women with FHA or menopause are eagerly anticipated. Further exploration is warranted to assess its viability as a prospective therapeutic target for clinical conditions related to bone metabolism such as osteoporosis, where current treatments have contraindications, frequently cause side-effects and are limited in duration.

## Future Avenues For Kisspeptin and NKB Research

In addition to the future research directions highlighted in the corresponding sections, we have included below some additional avenues for kisspeptin and NKB research and considerations of clinical applications of compounds targeting the kisspeptin/NKB pathways.

### Routes of kisspeptin administration

Kisspeptin presents significant diagnostic and therapeutic potential that could revolutionize the fields of reproductive endocrinology, metabolic and bone health. To date, current data have predominantly investigated subcutaneous or intravenous routes of administration, which may hinder its development as a diagnostic or therapeutic option. Exploring alternative administration methods, such as intranasal administration, could present an alternative non-invasive delivery route. Recent data in rodents have shown that up to 25% of the total GnRH population resides within the olfactory bulb (432). These GnRH neurons express olfactory and vomeronasal receptors activated by opposite-sex odors and maintain functional connections with olfactory and vomeronasal structures. Thus, GnRH neurons are well-equipped to detect and respond to social cues, such as pheromones, potentially impacting mating behaviors. Indeed, chemogenetic activation of GnRH neurons in the olfactory bulb of male mice increases the firing rate of GnRH neurons in the preoptic area, stimulating downstream LH and testosterone production (432). These findings suggest that the olfactory system could enable novel therapeutic approaches for modulating reproductive and behavioral functions, potentially through intranasal kisspeptin administration. Studies involving rodent and human intranasal kisspeptin-54 administration are currently underway, with promising preliminary results (433). In future, non-peptide agonists of the kisspeptin receptor could enable additional non-invasive routes of administration such as via the oral route.

### Clinical considerations for the bench to bedside translation of NKB antagonism

As tachykinins and their receptors are expressed in multiple extrahypothalamic tissues, antagonism of NKB action in the management of VMS could have additional effects beyond its thermoregulatory role. For instance, tachykinins are expressed within the enteric nervous system and within the mucosa of the gastrointestinal tract, albeit with marked interspecies variation (434). Data from animal studies demonstrated a role of tachykinins in gastrointestinal motility/peristalsis regulation, secreto-motor response and in the modulation of gastrointestinal inflammatory, immune, and sensory response. Thus, this has sparked interest in its therapeutic potential in gastrointestinal disorders. Conversely, the NK3R antagonist, Talnetant, did not demonstrate efficacy over placebo (435) in the control of irritable bowel syndrome (IBS) related discomfort, which did not corroborate fully with pre-clinical data. Moreover, Aprepitant and later Fosaprepitant (both NK1R antagonists), have been approved by the FDA in the treatment of chemotherapy-induced and postoperative emesis through its central actions on NK1 receptors (434,436). Overall, it will be informative to monitor for extrahypothalamic effects of NK3R antagonists, as well as the combination of NK3R and NK1R antagonists, currently being investigated in the management of menopausal VMS in the Phase 3 trials.

### Beyond vasomotor symptoms: Future areas of potential clinical applications targeting kisspeptin and NKB

Whilst vasomotor symptoms are the hallmark symptoms associated with the perimenopause and early postmenopausal years; women may experience a number of other symptoms that can negatively impact on quality of life. These include an altered metabolic rate that can be associated with weight gain (437), cognitive impairment (438), and mood disturbances (439). The changes in hypothalamic neuropeptide expression observed in post-mortem tissue from post-menopausal women and ovariectomized animal models raise the question if kisspeptin or NKB action contribute to these observed changes.

Rodent studies using global *Kiss1r* knockout models have consistently demonstrated that adult female *Kiss1r* knockout mice (at 18 weeks) exhibit an increase in body weight of up to 30% compared to wild-type controls (440–442), comprising mainly of increased adiposity (442). Notably, kisspeptin's effects on body weight appear to be sexually dimorphic, as adult male *Kiss1r* knockout mice show no significant changes in body weight (440,442). Some of the weight changes have been attributed to the hypogonadal state or inadequate sex-steroid replacement in *Kiss1r* knockout mice. However following ovariectomy, *Kiss1r* knockout mice had higher body weight, leptin levels, and adiposity compared to ovariectomized control females with similar sex-steroid milieu (440,442). Therefore, the effect of kisspeptin on energy homeostasis is likely mediated through direct effects on energy expenditure and indirectly through changes in reproductive hormone levels (440,442). In rats, a 25% gain in body weight was observed by three weeks post-ovariectomy, however this increase in body weight was abolished following selective ablation of the arcuate KNDy neurons (443). Furthermore, E2 replacement in control rats induced weight loss, an effect lost in KNDy ablated rats, thus suggesting the essential role of KNDy neurons in mediating the effects of E2 on body weight, at least in rats (443). However, at present corroborating data in humans is lacking, and the long-term effects of NK3R antagonism (which currently is entering clinical use for treatment of hot flashes) on body weight will be of interest.

Kisspeptin and its receptor are expressed in critical brain regions associated with memory and learning, including the amygdala and hippocampus (444). This anatomical localization supports the proposed cognitive roles of kisspeptin signaling. Experimental studies in mice suggest that stimulating kisspeptin signaling within the hippocampus can enhance learning and memory and exert neuroprotective effects, particularly concerning amyloid-β accumulation in the hippocampus, as observed in preclinical models of Alzheimer's disease (445). Kisspeptin-13 has been shown to improve memory consolidation in passive avoidance learning in male rodents (446) and enhance learning in zebrafish (447). Additionally, the administration of kisspeptin-13 via intracerebroventricular and intra-hippocampal routes in mice enhances memory retention and facilitates the formation of object and location recognition memory (448). These effects are abolished by the kisspeptin receptor antagonist, kisspeptin-234, indicating that kisspeptin signaling within the hippocampus is mediating these cognitive benefits (448).

Furthermore, preclinical studies on kisspeptin have reported a range of effects on anxiety, including anxiogenic (449–451), anxiolytic (452,453), and neutral outcomes (454). Central administration of kisspeptin to male rats also had no effect on other stress-related behaviors, including locomotion, sleep, and grooming (32). Acute administration of intravenous kisspeptin in clinical studies involving healthy heterosexual men (261), as well as studies involving men (267) and premenopausal women (264) with HSDD did not result in significant changes in psychometric measures of anxiety or circulating cortisol levels. In healthy men, kisspeptin administration led to reductions in negative mood as assessed by psychometric testing, mirroring findings seen in rodents (455), and enhanced activity in brain structures related to the reward system, such as the hippocampus, amygdala, and cingulate, in response to sexual images (261). Collectively, the evidence in humans suggests that kisspeptin might have antidepressant effects and a neutral impact on anxiety. Therefore, beyond its potential use in addressing symptoms in postmenopausal women, kisspeptin could potentially be used to treat patients experiencing mood disorders and sexual dysfunction, which often co-exist. Preclinical data on the NKB pathway on anxiety remains incongruous, with some studies suggesting pro-anxiety effects (456) and others indicating anxiolytic-like effects (457,458) in rodent models. Regarding depression, the NK3R agonist, aminosenktide, has demonstrated antidepressant activity in mice (459).

In conclusion, the potential roles of kisspeptin in modulating cognition, mood, and body weight represent a promising area for future research. A deeper understanding of these mechanisms may pave the way for targeted therapies aimed at alleviating some of the most challenging symptoms of menopause, as well as other psychosexual, mood, or cognitive conditions.

## Conclusion

Since the key physiological roles of kisspeptin and NKB in reproduction came to light in 2003, developments in pre-clinical and translational research have significantly advanced our understanding of the contributions of these two neuropeptides to the functioning of the HPG axis. The unique position of kisspeptin and NKB within the hypothalamic neuroendocrine circuitry has garnered significant interest for their potential applications in diagnosing and treating pubertal and reproductive disorders. Kisspeptin holds promise in distinguishing the causes of delayed puberty, while measurement of serum kisspeptin levels can aid in diagnosing precocious puberty and serve as marker for risk-stratification of pregnancy complications. Kisspeptin-based therapeutic avenues encompass various possibilities, including the restoration of pulsatile GnRH secretion in hypogonadal disorders like CHH, FHA, and hyperprolactinemia. Additionally, kisspeptin may serve as a safer oocyte maturation trigger in IVF treatment and offer a treatment option for distressing low sexual desire in HSDD.

Similarly, antagonism of the NKB pathway has emerged as a potential therapeutic target for uterine disorders including uterine fibroids and endometriosis, and for PCOS given their ability to reversibly suppress, but not abolish, the HPG axis. Most significantly, within the last year, we have seen the bench to bedside translation of NK3R antagonist, Fezolinetant, for treatment of hot flashes in menopause.

**Figure 10:**
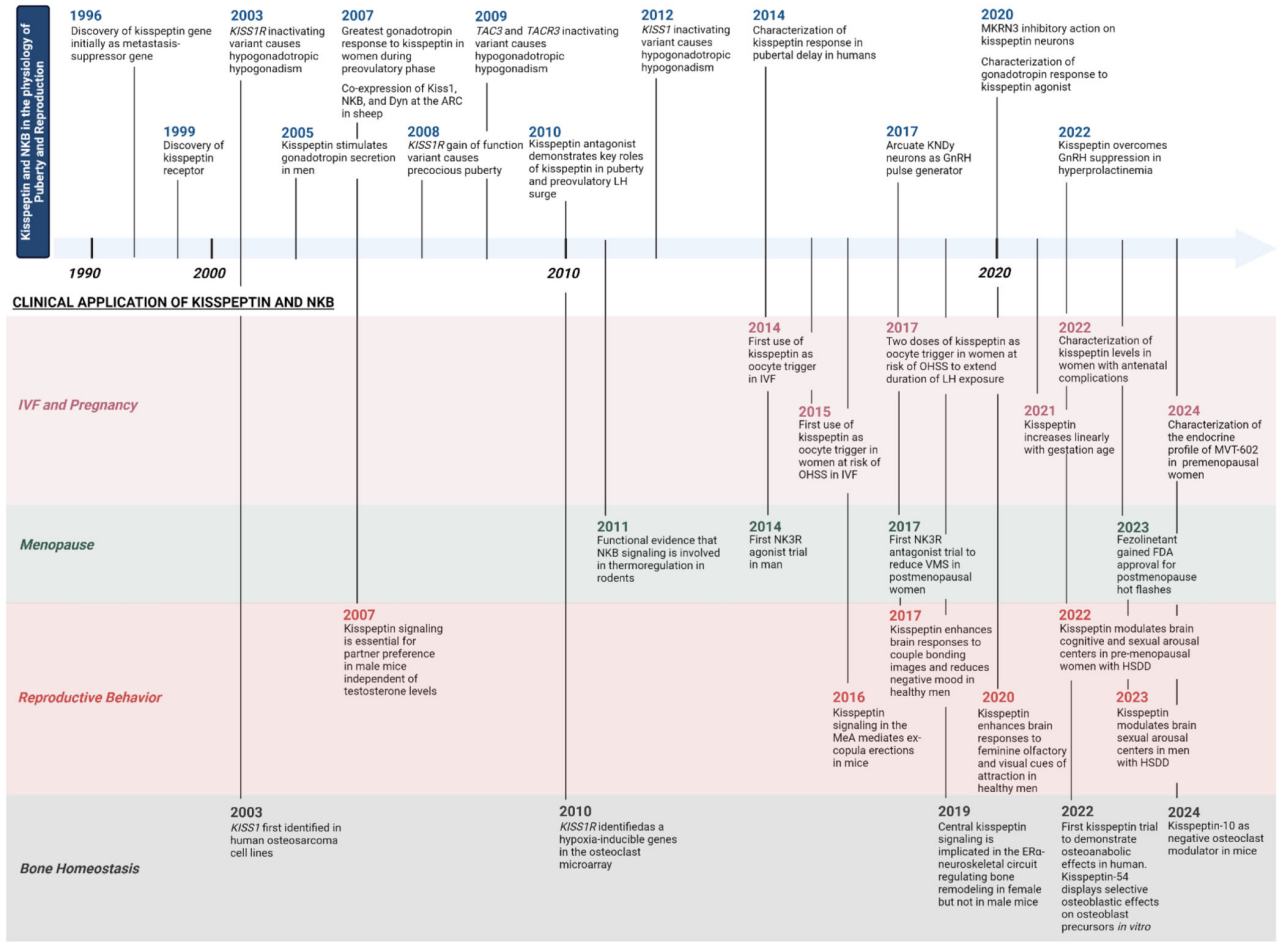
Timeline for key discoveries and clinical applications in the field of kisspeptin and neurokinin B research. ARC, arcuate nucleus; Dyn, dynorphin; FDA, Food and Drug Administration; GnRH, gonadotropin-releasing hormone; HSDD, hypoactive sexual desire disorder; IVF, *in vitro* fertilization; *KISS1*, kisspeptin gene in human; *Kiss1*, kisspeptin gene in non-human; *KISS1R*, kisspeptin receptor gene in human; KNDy, kisspeptin, neurokinin, dynorphin neuron; LH, luteinizing hormone; MeA, medial amygdala; MKRN3, makorin RING finger protein 3; NK3R, neurokinin 3 receptor; NKB, neurokinin B; OHSS, ovarian hyperstimulation syndrome; *TAC3*, neurokinin B gene in human; *TAC3R*, neurokinin 3 receptor gene in human; VMS, vasomotor symptoms. Figure created with BioRender.com.

Despite the impressive advancements over the past two decades (Figure 10) and the successful translation of the first-in-class NK3R antagonist, the reproductive biology of kisspeptin and NKB remains an intriguing research area with exciting potential therapeutic discoveries. The rapid technological advancements in optogenetics, fiber photometry and *in vivo* recordings of KNDy neuronal activity in freely moving animals have provided detailed insights into KNDy neurons at a cellular level. The identification of kisspeptin neurons in the human rostral hypothalamus and the positive estrogenic regulation of this neuronal population challenge long-held views regarding estrogen feedback in humans. Advancements in genetics and epigenetics have revealed novel silencers and activators that fine-tune pubertal timing through activation or repression of kisspeptin gene expression. Additionally, the integration of metabolic homeostatic cues and the involvement of afferent, intermediary neurons have deepened our understanding of the intricate connections between metabolic states and the HPG axis, which hold broader physiological implications beyond reproductive physiology.

In the field of reproductive endocrinology, significant unmet treatment challenges persist including therapeutic options for hypogonadotropic hypogonadism (FHA). There is also a pressing need for oocyte maturation trigger with favorable OHSS safety profile and new osteoporosis treatments. Addressing and understanding the distressing low sexual desire as seen in HSDD remains a challenge, while ongoing development and evaluation of different NK3R antagonists in menopausal hot flashes will provide data on their long-term safety and efficacy profile in different patient groups. These challenges are driving ongoing translational research efforts, particularly focusing on the kisspeptin and NKB pathways, paving the way for more advancements in the years to come.

## Clinical Highlights

Kisspeptin and neurokinin B (NKB) are hypothalamic neuropeptides that are indispensable for the healthy function of the hypothalamic-pituitary-gonadal (HPG) axis. Over the last two decades, preclinical and translational studies have consolidated the key roles of kisspeptin and NKB in the orchestration of reproductive function including acquisition and maintenance of reproductive capacity. Insights into the physiology of kisspeptin and NKB in reproduction, puberty, adult reproductive health including reproductive/sexual behavior, pregnancy, bone health and menopause, have facilitated the translational application of agents targeting the kisspeptin and NKB pathways as potential treatments for reproductive disorders. Clinical studies investigating kisspeptin have suggested clinical potential for its use in triggering oocyte maturation during *in vitro* fertilization (IVF) treatment, in the restoration of pulsatile secretion of GnRH in functional hypothalamic amenorrhea, as a biomarker with the potential to predict pregnancy complications such as miscarriage, as a diagnostic test of pubertal disorders, as well as holding potential for the treatment of hypoactive sexual desire disorder (HSDD), and osteoporosis. Blockade of NKB pathways has potential utility in the treatment of hyperandrogenism due to polycystic ovary syndrome (PCOS), and is now available in the clinic for the relief of menopausal vasomotor symptoms.
